# Prognostic and clinicopathological insights of phosphodiesterase 9A gene as novel biomarker in human colorectal cancer

**DOI:** 10.1186/s12885-021-08332-3

**Published:** 2021-05-20

**Authors:** Tasmina Ferdous Susmi, Atikur Rahman, Md. Moshiur Rahman Khan, Farzana Yasmin, Md. Shariful Islam, Omaima Nasif, Sulaiman Ali Alharbi, Gaber El-Saber Batiha, Mohammad Uzzal Hossain

**Affiliations:** 1 Department of Genetic Engineering and Biotechnology, Faculty of Biological Science and Technology, Jashore University of Science and Technology, Jashore, 7408 Bangladesh; 2 Department of Fermentation Engineering, School of Biotechnology, Jiangnan University, Wuxi, China; 3 Department of Reproductive and Developmental Biology, Graduate School of Life Science, Hokkaido University, Sapporo, 5 Chome Kita 8 Jonishi, Kita Ward, Sapporo, Hokkaido 060-0808 Japan; 4 Department of Biology, University of Kentucky, 101 T.H. Morgan Building, Lexington, KY 40506-022 USA; 5 Department of Physiology, College of Medicine, King Saud University [Medical City], King Khalid University Hospital, PO Box 2925, Riyadh, 11461 Saudi Arabia; 6 Department of Botany & Microbiology, College of Science, King Saud University, P.O Box 2455, Riyadh, 11451 Saudi Arabia; 7 Department of Pharmacology and Therapeutics, Faculty of Veterinary Medicine, Damanhour University, Damanhour, AlBeheira 22511 Egypt; 8 Bioinformatics Division, National Institute of Biotechnology, Ganakbari, Ashulia, Savar, Dhaka 1349 Bangladesh

**Keywords:** PDE9A gene, Colorectal cancer, Methylation, Pathways, Biomarker

## Abstract

**Background:**

PDE9A (Phosphodiesterase 9A) plays an important role in proliferation of cells, their differentiation and apoptosis via intracellular cGMP (cyclic guanosine monophosphate) signaling. The expression pattern of PDE9A is associated with diverse tumors and carcinomas. Therefore, PDE9A could be a prospective candidate as a therapeutic target in different types of carcinoma. The study presented here was designed to carry out the prognostic value as a biomarker of PDE9A in Colorectal cancer (CRC). The present study integrated several cancer databases with in-silico techniques to evaluate the cancer prognosis of CRC.

**Results:**

The analyses suggested that the expression of PDE9A was significantly down-regulated in CRC tissues than in normal tissues. Moreover, methylation in the DNA promoter region might also manipulate PDE9A gene expression. The Kaplan–Meier curves indicated that high level of expression of PDE9A gene was associated to higher survival in OS, RFS, and DSS in CRC patients. PDE9A demonstrated the highest positive correlation for rectal cancer recurrence with a marker gene CEACAM7. Furtheremore, PDE9A shared consolidated pathways with MAPK14 to induce survival autophagy in CRC cells and showed interaction with GUCY1A2 to drive CRPC.

**Conclusions:**

Overall, the prognostic value of PDE9A gene could be used as a potential tumor biomarker for CRC.

**Supplementary Information:**

The online version contains supplementary material available at 10.1186/s12885-021-08332-3.

## Introduction

Cancer is the second most common cause of death worldwide which occurs due to the uncontrolled growth of cells that can migrate to other parts of the body [1, 2]. According to WHO-Cancer Report-2020-Global Profile, around 18 million new cancer cases and 9.6 million deaths are estimated worldwide from cancer in 2018. Therein, Colon cancer (CRC) is imputed as the third most diagnosed cancer in the world [3]. Therefore, CRC is described as a malignant tumor that arises from the inner surface of the large intestine [4]. Over 50% of patients die from colon cancer despite having intensive investigations and therapeutic improvements [5]. However, the main reason behind the death can be pointed as a result of spreading the uncontrollable cell division tendency to other organs spontaneously [6]. CRC is known as westernized diseases and it shows comparatively lower lethality in the South Asian population than the western countries [3, 7]. WHO reported in 2018 that, Colon cancer deaths in Bangladesh are 3598, or 0.46% of total deaths (https://www.worldlifeexpectancy.com/). According to Syed Akram Hussain and Richard Sullivan, colon cancer is one of the 10 common causes of deaths from cancer in both males and females [8]. But the affected case of CRC is expected to increase by 60% to more than 2.2 million new cases and 1.1 million deaths by 2030 [9]. Besides, epidemiological studies reveal that CRC is related to familial and hereditary factors, besides some other factors like age, environment, and lifestyle also affect [10–12]. Experiments on CRC patients show that DNA methylation and covalent modification of histones results in carcinogenesis [13]. So, early identification of CRC is extremely important for the prevention of CRC [14]. Perrectum examination is the simplest way to CRC recognition [15]. However, the most efficient and commonly applied method of CRC recognition is endoscopy [15]. Molecular diagnosis methods for CRC, based on genetic and epigenetic tests, also have limited applicability [16, 17]. Stool DNA (sDNA) is very stable which can indicate the changes in DNA in colorectal adenocarcinomas (COAD) [15]. All of these instigate to find the unknown mechanisms contributing to CRC malignancy and the necessity for the development of potential biomarkers for the CRC prognosis.

PDEs (Cyclic nucleotide phosphodiesterases) consisted of a large superfamily of 11 PDE gene families (PDE1 to PDE11) and involves in regulating the intracellular levels of the second messengers cAMP and cGMP [18]. PDE9A is a member of eleven PDE isoforms located at q22.3 position of the 21st chromosome in the human cell [19]. PDE9A encodes high-affinity cGMP-Specific 3′,5′-Cyclic Phosphodiesterase 9A protein which hydrolyzes the second messenger cGMP, that regulates many important physiological processes [20–22]. The regulation and expression of PDEs (cyclic nucleotide phosphodiesterases) play a significant role in tumor progression and inhibition [23]. Many hematological malignancies and carcinomas have been connected with decreased levels of cAMP and/or cGMP by the PDE gene family [24]. PDE2A, PDE8B, and PDE11A expression are involved with adrenocortical tumors [25], high expression of PDE4 has been detected in brain tumors [26], in glioblastoma multiforme PDE5 is strongly expressed [27], and increased PDE7B expression found in Chronic lymphocytic leukemia [28]. The cDNA of PDE9A was coined in 1998 and noted as the ninth member of the PDEs family [29]. Only from the PDE9A gene, twenty-eight splice variants can arise (https://asia.ensembl.org/). These can create more proteins through alternative splicing of mRNA or multiple promoters and transcription start sites [18]. Although PDE9 mRNA has been detected in many organs; but, the highest expression has been noted in prostate, colon, small intestine, brain, kidney, spleen, thymus, and hematopoietic cells [22]. The protein expression of PDE9A is highly conserved and also associated with prostate cancer [30]. DNA hypermethylation of the PDE9A gene results in reduced mRNA expression in tumorous tissues [31]. Thus, our investigation was designed to determine the prognostic and clinicopathological importance of the PDE9A gene in CRC since there is no obvious report regarding its surveillance.

The most difficult job for an oncologist is to evaluate the prognostic significance of a gene which is also an essential skill for treatment information [32]. Cancer bioinformatics is believed to be a way of early evaluation of a cancer which is used to identify the role of a gene for any cancer before going laboratories. Various database provides gene expression and related information for analysis in various cancer types like UALCAN provides information on pan-cancer molecular subtypes [33]. Using these onco-informatics processes it was found that DNA topoisomerases expression in lung cancer was higher than the normal tissues [34]. Ping Yan *et al*., recommended that COL1A1, MMP2, FN1, TIMP1, SPARC, COL4A1, and ITGA5 may be potential biomarkers and therapeutic targets for GC by adopting in silico process [35].

In this study, our target is to assess and identify the role of phosphodiesterase 9A (PDE9A) gene which can portray a complete concept to understand the expression and clinical significance in CRC. Therefore, we investigate the patterns of expression, promoter methylation patterns of our target gene. We also explore the combined prognostic relevance, the association of genes co-expressed with PDE9A, and the interaction network of PDE9A in CRC. Moreover, this multi-omics data mining approach can provide useful intimation to instigate the researcher for finding new approaches in anti-cancer therapies.

## Materials and methods

### Expression analysis of PDE9A

To retrieve the mRNA expression of the PDE9A gene in various cancer types, we have employed Oncomine database (http://www.oncomine.org/) that investigates differential expression patterns of a gene in diverse clinical cancer specimens and analogous normal controls, incorporates 715 independent datasets and 86,733 samples [36, 37]. The threshold parameters were settled as reflects: keywords, PED9A gene, primary filter, cancer vs. normal analysis; cancer type, Colorectal Cancer, data type: mRNA, fold change: 2, *P*-value: 1E− 4, gene ranking: 10%. Co-express genes with PDE9A were also investigated for further analysis. Thereafter, we used UALCAN database (http://ualcan.path.uab.edu/index.html) to analyze expression, survival, methylation, pan-cancer view, and correlation data [38, 39]. PDE9A gene expression and methylation were analyzed based on sample types, individual cancer stages, patient’s race, patient’s weight, patient’s gender, patient’s age, histological subtype, nodal metastasis status, and TP53 mutation status. The positive correlation between PDE9A and CEACAM7 genes for Colon adenocarcinoma (COAD) was assessed and Pearson’s correlation analysis value was 0.54. The *p*-value was considered statistically significant when it was less than 0.05 (*p* < 0.05) for all the results. The screening parameters were set: “Gene: PDE9A”; “Analysis Type: TCGA Gene analysis”; “Cancer Type: Colon adenocarcinoma”; “Data Type: TCGA dataset”. OncoLnc (http://www.oncolnc.org/) online tools evaluate survival correlations with expression data and retrieving clinical data for mRNAs, miRNAs, and lncRNAs (long non-coding) [40]. OncoLnc database gives the results of the Cox analysis based on 21 cancer studies for 8647 patients from that link to TCGA survival data or MiTranscriptome beta lncRNA data [41]. This webtool analysis Cox regression results for PDE9A gene and provides information on cox coefficient, Rank, Median expression, *P*-value, Mean expression, FDR corrected, and Kaplan-Meier plot against Colon adenocarcinoma (COAD). The value 25 was inputted for Lower percentile and Upper percentile to generate Kaplan Meier plot. PrognoScan Database (http://dna00.bio.kyutech.ac.jp/PrognoScan/) is a cancer microarray dataset that provides information for analyzing potential therapeutic value that could accelerate in silico cancer research by clinical annotation [42].

### Clinicopathological insights of PDE9A

Gene Expression database of Normal and Tumor tissues 2 (GENT2) (http://gent2.appex.kr/gent2/) was utilized to retrieve the information of PDE9A in tissue wide expression pattern, meta-survival analysis, and prognostic significance of a gene of interest-based on tumor subtypes [43, 44]. Accordingly, we put the PDE9A gene for tissue wide gene expression patterns across 72 paired tissue. Subtype profiling along with durability analysis in colon tissue for the PDE9A gene was also explored where results were shown in the box and dot-plot of individual subtype. Moreover, progression-free survival in colon tissue for the PDE9A gene was evaluated by meta-analysis of analytical tests (t-test, log2 fold changes, etc.). UCSC Xena (https://xena.ucsc.edu/), a high-performance data mining platform to visualize and analyze functional genomic data sets for both enormous public archives (TCGA, GDC, etc) and private datasets between genomic and phenotypic variables [45] was employed to set up a correlation heat map between PDE9A and CEACAM7 gene expression in the same patient cohort through data processing in TCGA (The Cancer Genome Atlas Program) colon cancer (COAD). Subsequently, PDE9A DNA methylation status is also examined by adopting this UCSC Xena portal [46]. The matched 551 TCGA Colon Cancer specimens were wielded for all analyses. The clinical features of PDE9A was explored by the utilization of cBioPortal for Cancer Genomics (https://www.cbioportal.org/) which is an open-source, open-access web-based data repositories that explore, visualize, and analyze multidimensional cancer genomics data from TCGA [47, 48]. Two TCGA datasets of COAD, particularly “TCGA Nature 2012 (276 samples)” and “TCGA PanCancer Atlas (594 samples) were culled for further investigation of PDE9A gene alterations or copy number alterations (CNA) using the cBioPortal database. The OncoPrint, cancer types summary, plots, mutations, survival, copy number segment, pathways, expression tabs were guided following the default settings of the cBioPortal.

### Determination of prognostic effects

Gene Expression Profiling Interactive Analysis (GEPIA) (http://gepia.cancer-pku.cn/index.html) was used to explore the differential expression analysis and also to determine the prognostic effects on COAD cancer patients [49]. GEPIA facilitates user’s data mining in diverse areas including differential expression analysis in cancer vs. normal samples, profiling plotting according to cancer categories or various pathological phases, patient survival analysis, correlation analysis, identical gene disclosure, and dimensionality reduction analysis [50].

### Co-expression and interacting network analysis

GeneCards, the human gene database (https://www.genecards.org/) is a searchable, speedy, and sophisticated user-friendly integrative database that furnishes information of all annotated and predicted human genes [51, 52]. For the, GeneCards was scrutinized to retrieve the information of PDE9A gene regarding the gene functions, pathways, and interactions, mRNA expression in normal human tissues, SNPs, disorders associated with this gene, etc. Later, the R2 platform (http://r2platform.com) was utilized the R2 web interface to explore the PDE9A gene expression, correlation analysis between PDE9A and CEACAM7 genes as well as to generate a Kaplan-Meier survival plot. R2 platform generates Kaplan-Meier plot (overall survival) for a specific dataset and the Kaplan-Meier plot was generated for PDE9A gene against Tumor Colon Adenocarcinoma - TCGA - 286 - rsem - tcgars dataset with the optimum cut-off values. The co-expression between PDE9A and CEACAM7 genes was identified in Tumor Colon Adenocarcinoma - TCGA - 286 - rsem - tcgars dataset. This dataset provides data analyzing 286 samples and source: TCGA ID: COAD with date: 2000-01-01.

### Functional insights of PDE9A

GeneMANIA (http://www.genemania.org/) web address predicts the function of a gene or gene lists and identifies the physical interaction, genetic interactions, co-expression, pathway, co-localization, and shared protein domain [53]. We used GeneMANIA database to obtain an interaction network of related connected genes for PDE9A. Genes can be connected by the interacted network based on different attributes. Here nodes represent genes whereas links show networks. The screening status was set: Organism: *Homo sapiens*; Query Gene: PDE9A; Maximum Resultant Gene: 25; Maximum Resultant Attributes: 10; Query-Dependent Weighting: Automatically and rest are used as default. Furtheremore, STRING database (https://string-db.org/) was also employed to predict the protein-protein interactions including direct (physical) and indirect (functional) associations [54, 55]. The amino acid sequence used in STRING database was retrieved from KEGG database (593 aa) and *Homo sapiens* were selected as initial parameters.

## Results

**Fig. 1:**
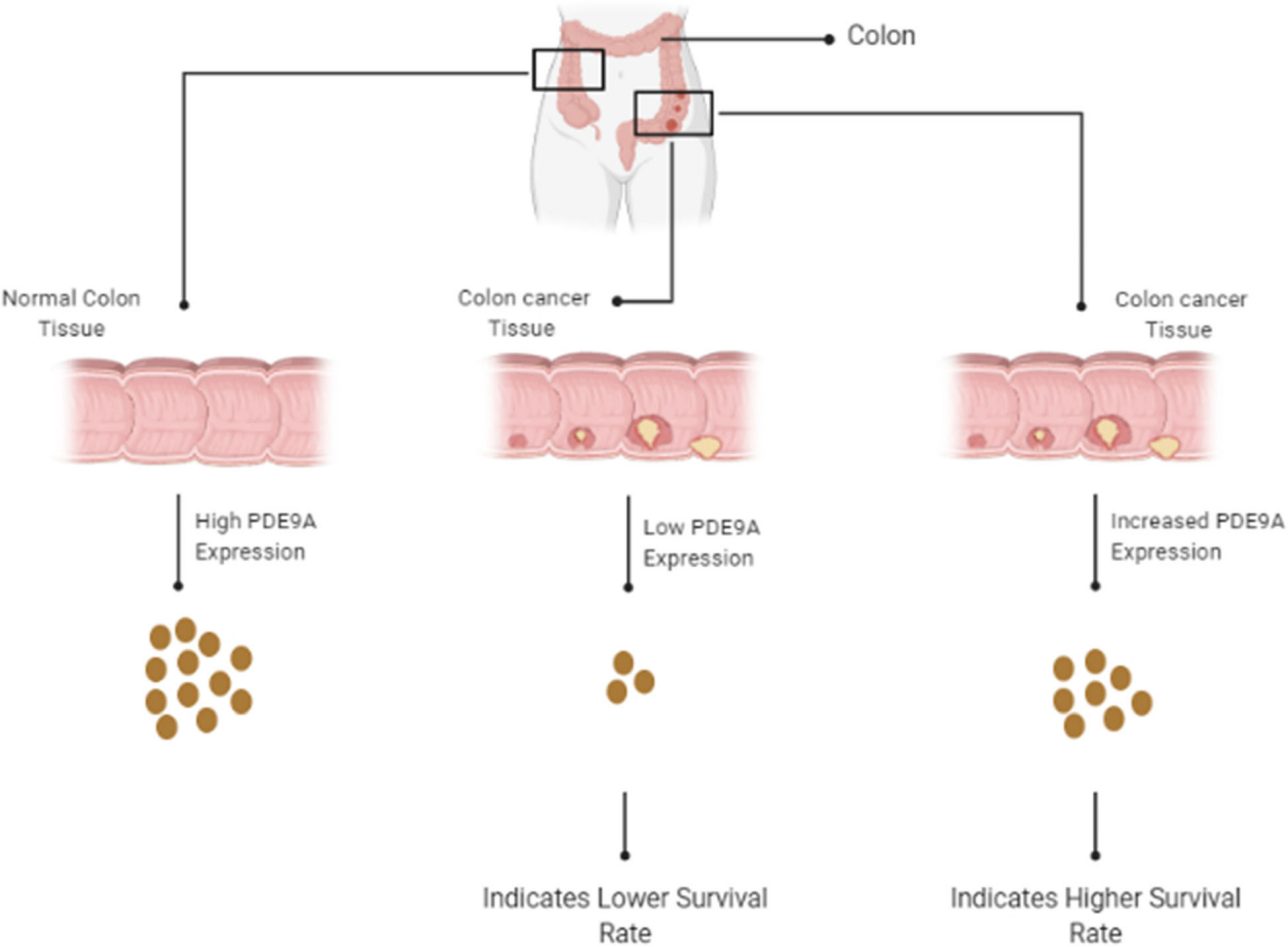
Graphical demonstration of PDE9A gene expression and their relations with patients survivability in colorectal cancer tissues

The workflow of the study is shown in Fig. 1.

### PDE9A mRNA expression level evaluation in various cancer

**Fig. 2:**
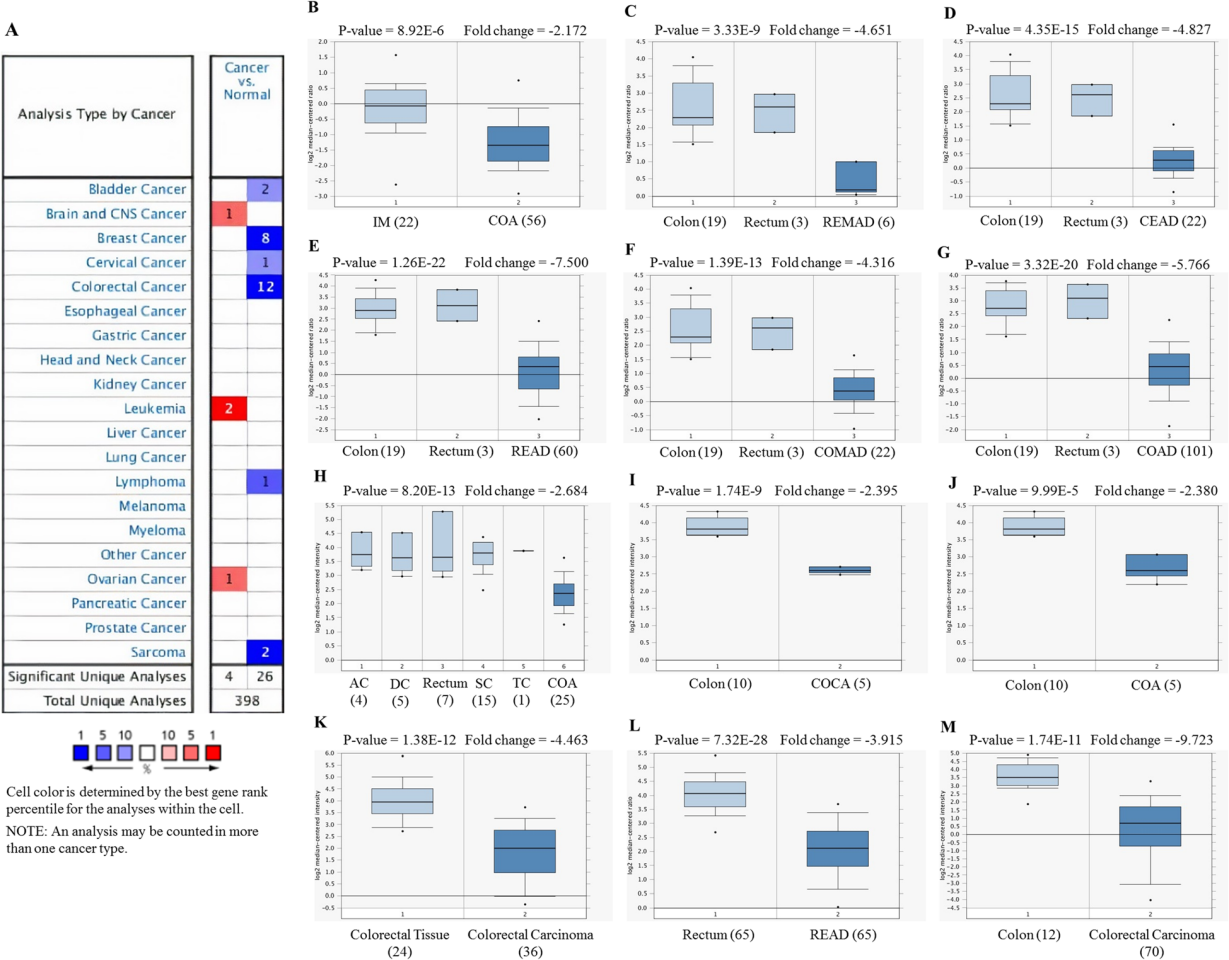
**a** Expression of PDE9A in different cancers from Oncomine database with significant mRNA depending on different types of cancer and corresponding normal tissue. Here, upregulation is marked in red and downregulation is blue. The threshold parameters were set as follows: *P*-value of 1E− 4, fold change 2, gene ranking 10%. **b-m** box plot comparing PDE9A expression in different colorectal cancer types (normal tissue in left and cancerous tissue in right) depicted significant downregulation of PDE9A in **b** Colorectal Adenoma (COA), **c** Rectal Mucinous Adenocarcinoma (REMAD), **d** Cecum Adenocarcinoma (CEAD), **e** Rectal Adenocarcinoma (READ), **f** Colon Mucinous Adenocarcinoma (COMAD), **g** Colon Adenocarcinoma (COAD), **h** Colon Adenoma (COA), **i** Colon Carcinoma (COCA), **j** Colon Adenoma (COA), **k** Colorectal Carcinoma, **l** Rectal Adenocarcinoma (READ), **m** Colorectal Carcinoma

**Fig. 3:**
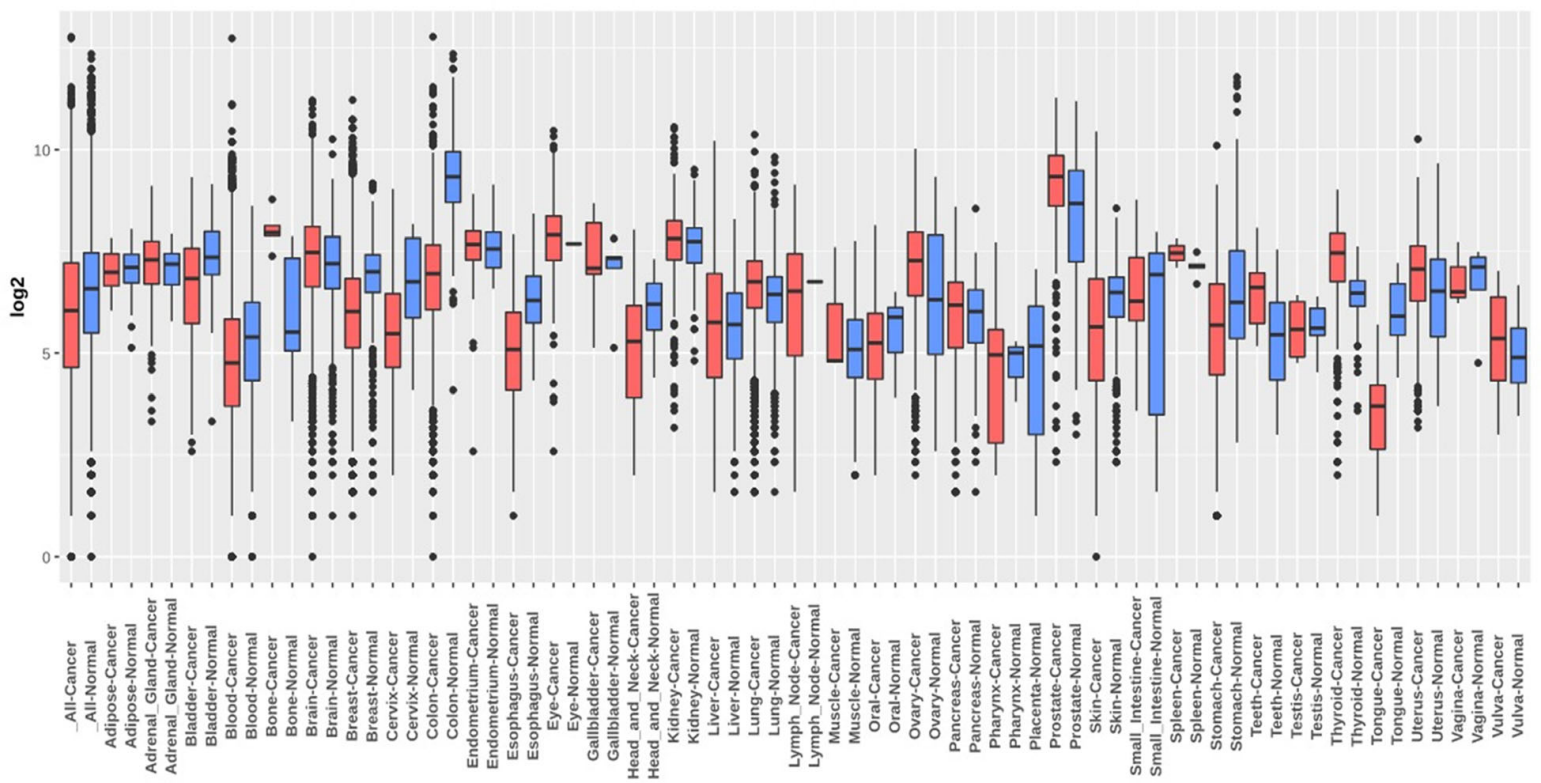
PDE9A mRNA expression level across various cancer types was obtained from GENT2 database in normal and tumor tissues. Red color designates a boxplot of cancer samples whereas blue specifies a boxplot of normal samples

**Fig. 4:**
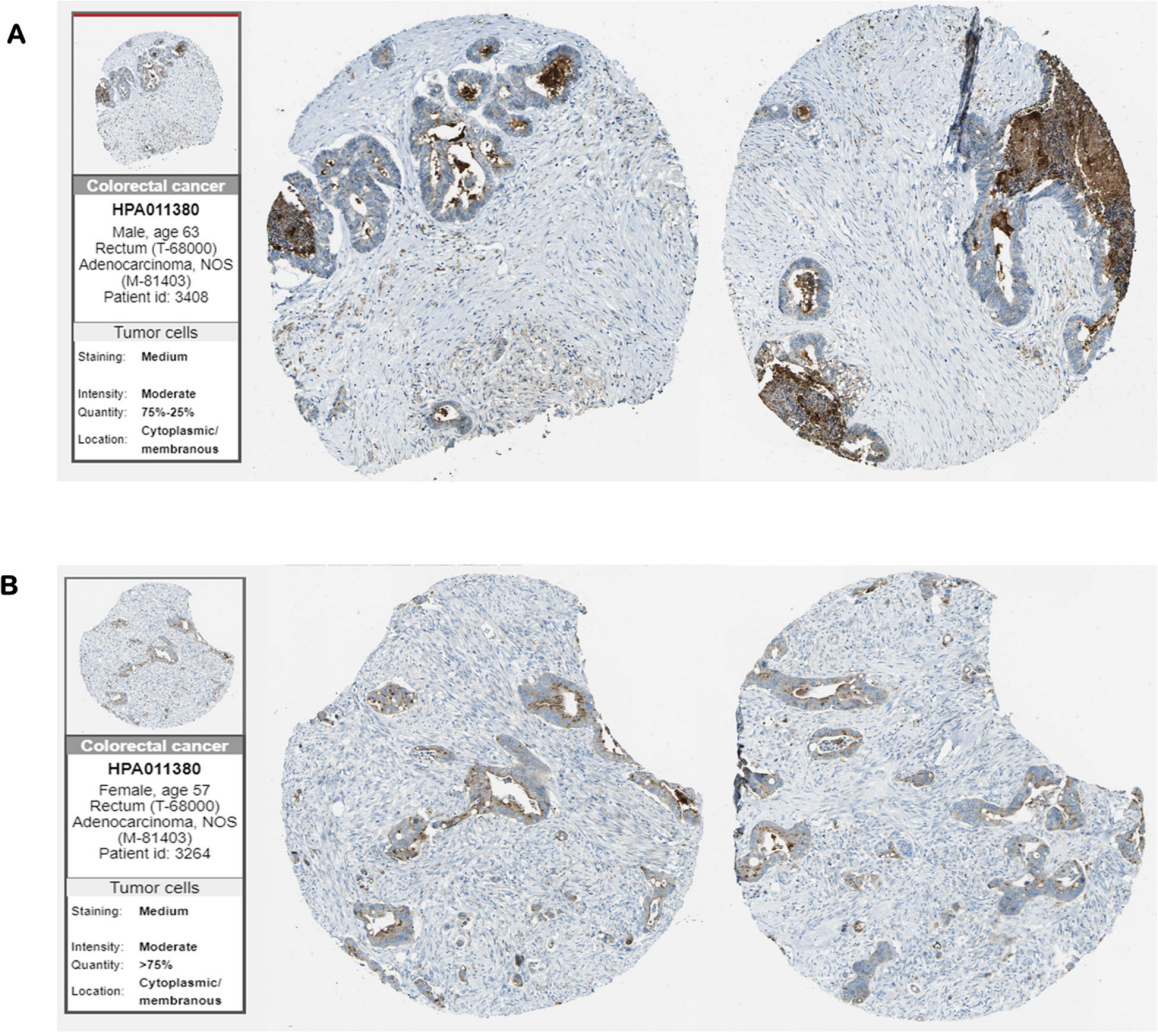
The figure represents antibody staining immunohistochemical images of PDE9A protein expression in colorectal cancer tissues by the Human Protein Atlas website (**a**) sample of a male patient aged 63, (**b**) sample of a female patient aged 57

For analyzing the expression of PDE9A in various cancers, we compared its transcriptional levels in various cancer tissues with their corresponding normal tissues by adopting various databases like Oncomine, GEPIA, Gent2, UALCAN, GeneCard. Using the differential analysis tool of the Oncomine database, we analyzed cDNA microarray data for PDE9A in 20 different human cancer types. Significant mRNA upregulation (red) or downregulation (blue) was depicted by the Oncomine graphical results. According to Oncomine, PDE9A was discovered to be downregulated in various colorectal cancer including Colorectal Adenoma, Rectal Mucinous Adenocarcinoma, Cecum Adenocarcinoma, Colon Adenoma, Rectal Adenocarcinoma, Colorectal Carcinoma, Colon Mucinous Adenocarcinoma, Colon Adenocarcinoma, Colon Carcinoma (Fig. 2, Table 1). The expression of PDE9A was also estimated by the GENT2 database where results are depicted in boxplot across 72 paired cancer vs. normal tissues. GENT2 database reveals that PDE9A expression is downregulated in the bladder, blood, breast, cervix, colon, skin, esophagus, head and neck, oral, stomach, and tongue cancers, and is upregulated in various cancer including adrenal gland, bone, brain, ovary, prostate, teeth, thyroid, uterus, and vulva cancer through the GLP570 platform (Fig. 3). UALCAN website also provides expression information of PDE9A across TCGA cancers in which bladder, breast, colon, kidney, rectal, and stomach cancers expression are lower and cholangiocarcinoma, glioblastoma, lung, prostate, thyroid cancers expression are higher following their corresponding normal samples (Supplementary Fig. 1A and 1B). To confirm the result attained from Oncomine, GENT2, UALCAN database, we performed PDE9A expression analysis using another platform, GEPIA. It provides results in the dot plot and bar plot. GEPIA reveals the expression profile across all tumor tissues and corresponding normal tissues (Supplementary Fig. 1C). mRNA expression of PDE9A in COAD was significantly lower in COAD compared to normal tissues. GeneCards database shows the location of the PDE9A gene in 21q22.3 cytogenetic band along with mRNA expression in normal human tissues based on RNAseq, Microarray, and SAGE (Serial Analysis of Gene Expression) (Supplementary Fig. 1D). The immunohistochemical staining images of PDE9A protein expression in CRC are given in (Fig. 4). Systematic mRNA expression analysis carried out here from multiple databases was enough to prove that PDE9A expression across a wide range of cancer types in which it is significantly downregulated in CRC.

**Table 1:** Expression of PDE9A among different subtypes of colorectal cancer and normal individuals using the Oncomine database

Dataset	Colorectal Cancer Subtype	*P* value	Fold change	*t* test	Rank (%)
Gaspar colon	Colorectal Adenoma (56)	8.92E-6	−2.172	−5.013	2
TCGA Colorectal	Rectal Mucinous Adenocarcinoma	3.33E-9	−4.651	−10.639	1
	Cecum Adenocarcinoma	4.35E-15	−4.827	−12.130	2
	Rectal Adenocarcinoma	1.26E-22	−7.500	−14.627	2
	Colon Mucinous Adenocarcinoma	1.39E-13	−4.316	−10.535	2
	Colon Adenocarcinoma	3.32E-20	−5.766	−16.269	3
Sabates-Bellver Colon	Colon Adenoma	8.20E-13	−2.684	−9.129	1
Skrzypczak Colorectal 2	Colon Carcinoma	1.74E-9	−2.395	−14.557	2
	Colon Adenoma	9.99E-5	−2.380	−7.633	9
Skrzypczak Colorectal	Colorectal Carcinoma	1.38E-12	−4.463	− 8.807	2
Gaedcke Colorectal	Rectal Adenocarcinoma	7.32E-28	−3.915	−14.541	3
Hong Colorectal	Colorectal Carcinoma	1.74E-11	−9.723	−9.735	4

### mRNA expression and methylation status of the PDE9A gene with various clinicopathological parameters in CRC patients

**Fig. 5:**
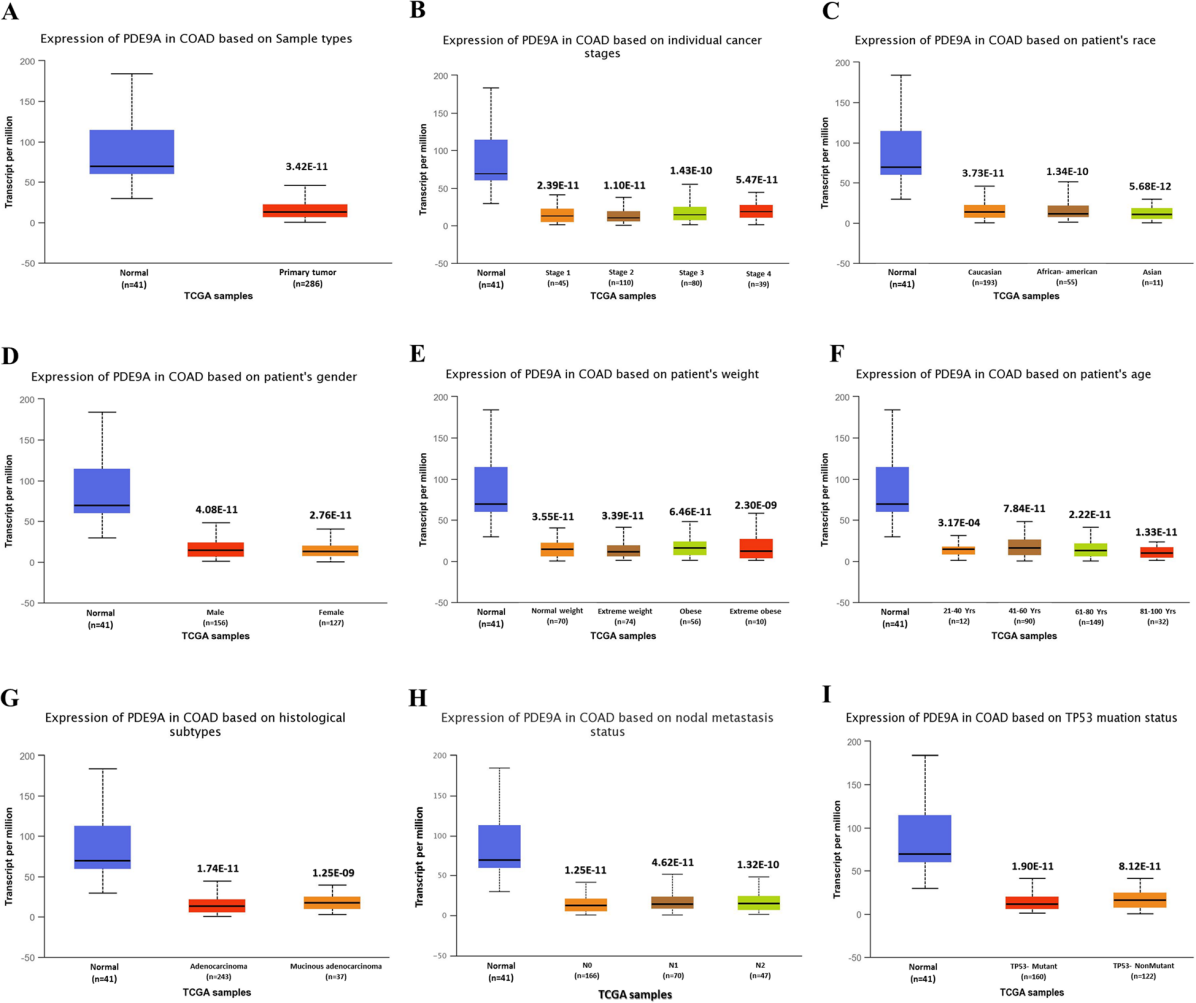
mRNA expression analysis of PDE9A based on different variables for Colon Adenocarcinoma (COAD) patients. Expression of PDE9A in COAD based on (**a**) sample types, (**b**) individual cancer stages, (**c**) patient race, (**d**) patient gender, (**e**) patient weight, (**f**) patient weight, (**g**) tumor histological subtype, (**h**) nodal metastasis status, and (**i**)T53 mutation status

**Fig. 6:**
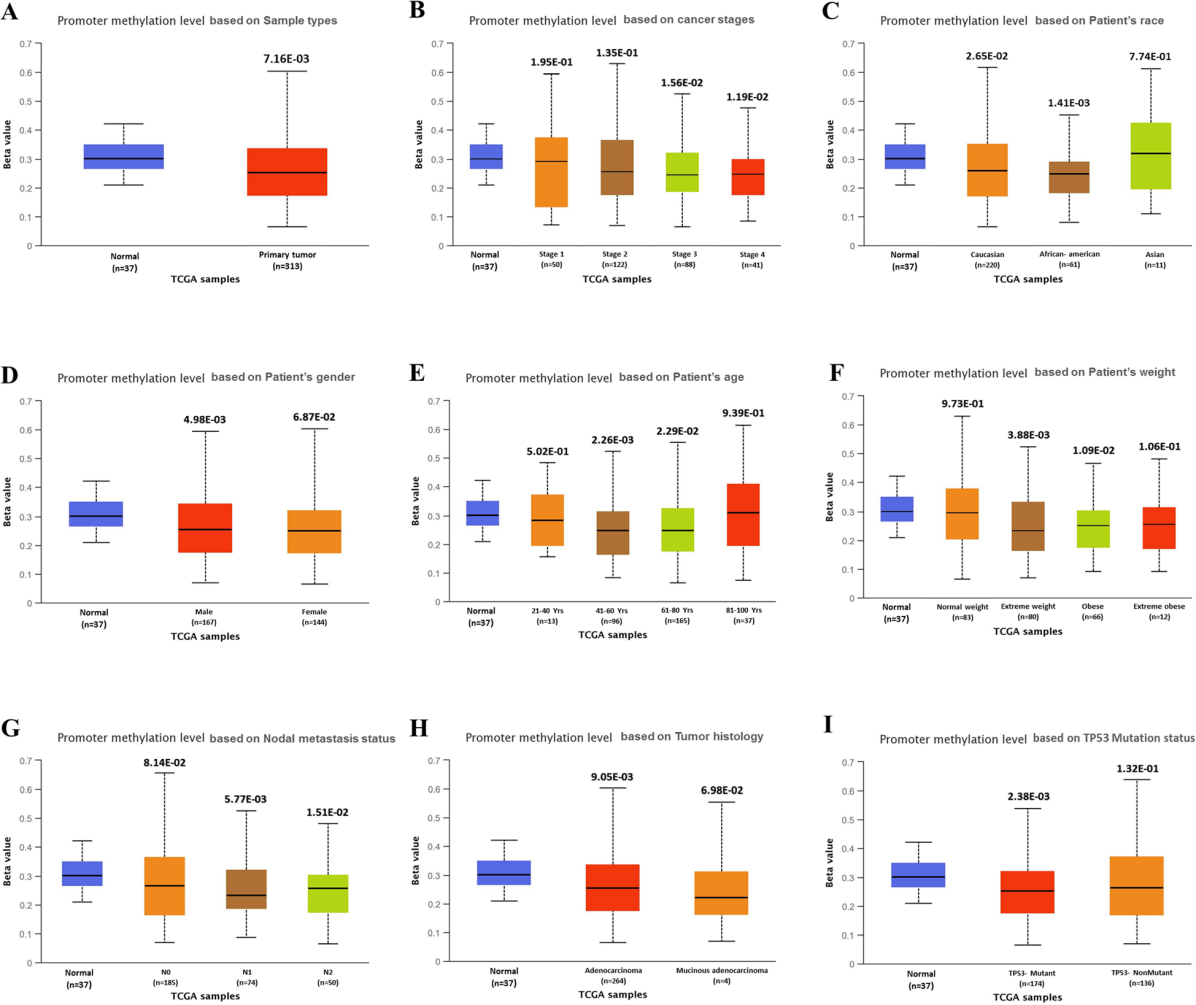
PDE9A promoter methylation profile based on different variables of TCGA colon adenocarcinoma (COAD) from UALCAN database. The variables are (**a**) sample types, (**b**) individual cancer stages, (**c**) patient’s race, (**d**) patient’s gender, (**e**) patient’s age, (**f**) patient’s weight, (**g**) nodal metastasis status, (**h**) histological subtype, and (**i**) TP53 mutation status. According to UALCAN, the Beta value indicates the level of DNA methylation ranging from 0 (unmethylated) to 1 (fully methylated). Different beta value cut-off has been considered to indicate hypermethylation [Beta value: 0.7–0.5] or hypo-methylation [Beta-value: 0.3–0.25]

**Fig. 7:**
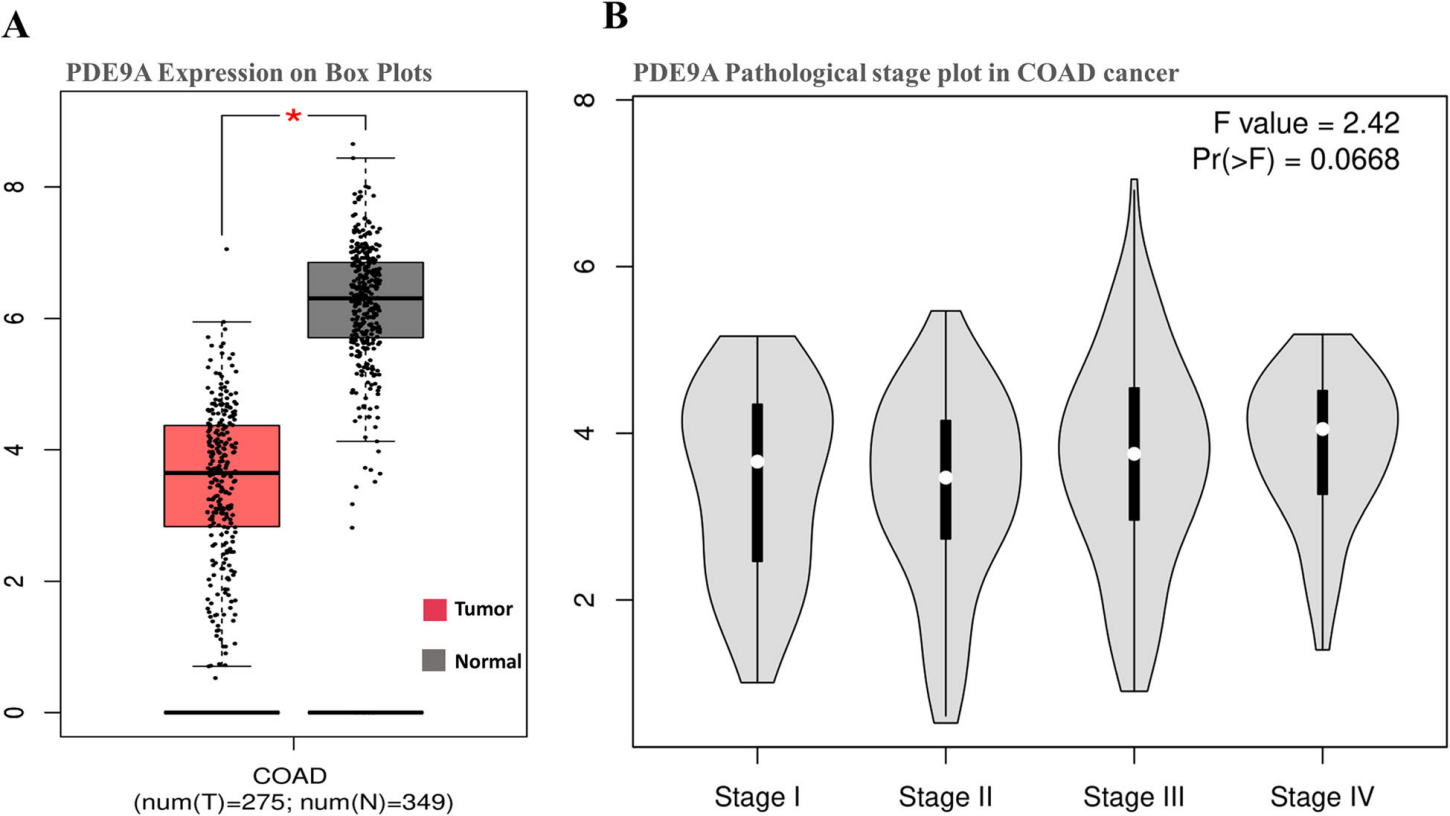
**a** Box plot of PDE9A mRNA expression using GEPIA database in Colon Adenocarcinoma (COAD) patients, **b** The connection between PDE9A expression with different tumor stages in patients with COAD from GEPIA database

Based on different clinicopathological parameters, the UALCAN database has been employed to analyze the expression pattern and methylation status of the PDE9A gene in COAD. Statistical analysis from this database revealed that the expression of PDE9A is significantly attenuated in colon cancer samples compared with the normal counterparts. The connection between PDE9A expression and different clinicopathological characteristics in CRC samples exhibits that PDE9A is down-regulated in all the variables (sample types, individual cancer stages, patient’s race, patient’s weight, patient’s gender, patient’s age, histological subtype, nodal metastasis status, and TP53 mutation status) used here compared to the normal (Fig. 5 and Supplementary Table 1). Next, promoter methylation of PDE9A had been analyzed as it is thought that epigenetic modulation is the key factor to reduce mRNA expression and methylation levels in the promoter regions. Thus methylation is closely associated with the development of tumors. The PDE9A promoter methylation had been analyzed in colon cancer tissues. It was found that the promoter methylation level of PDE9A in sample types, individual cancer stages, patient race, patient gender, patient age, patient weight, tumor histology, and T53 mutation status was reduced than normal tissues (Fig. 6 and Supplementary Table 2). Furthermore, the correlation analysis between PDE9A expression and DNA methylation heat map in the TCGA colon cancer sample types depicted that PDE9A expression positively related to PDE9A promoter methylation at some CpG sites (blank frame) (Supplementary Fig. 2). Hence, the results implied that the status of methylation in the PDE9A DNA promoter region can be related to PDE9A expression in cancer tissues. Besides, the GEPIA database was also employed to identify mRNA expression of PDE9A between COAD samples and normal tissue samples. The PDE9A expression is significantly lower or downregulated (significance; *P* > 0.05) in COAD than compared with normal tissues (Fig. 7a). The relatedness between PDE9A mRNA expression levels and different tumor stages of COAD was also evaluated, where PDE9A is significantly downregulated in stage II (Fig. 7b). To establish our expression concept strongly in COAD, we further used the Gent2 database to add COAD subtype profiling for PDE9A. The database provides five Subtypes (Molecular subtype, AJCC Stage, Duke Stage, Grade, and Histology) for colon cancer analyzing 1146 samples. The PDE9A gene expression in colon cancer tissue based on different subtypes are shown in Supplementary Fig. 3 via box and dot-plot.

### Survival plot analysis and prognostic value

**Fig. 8:**
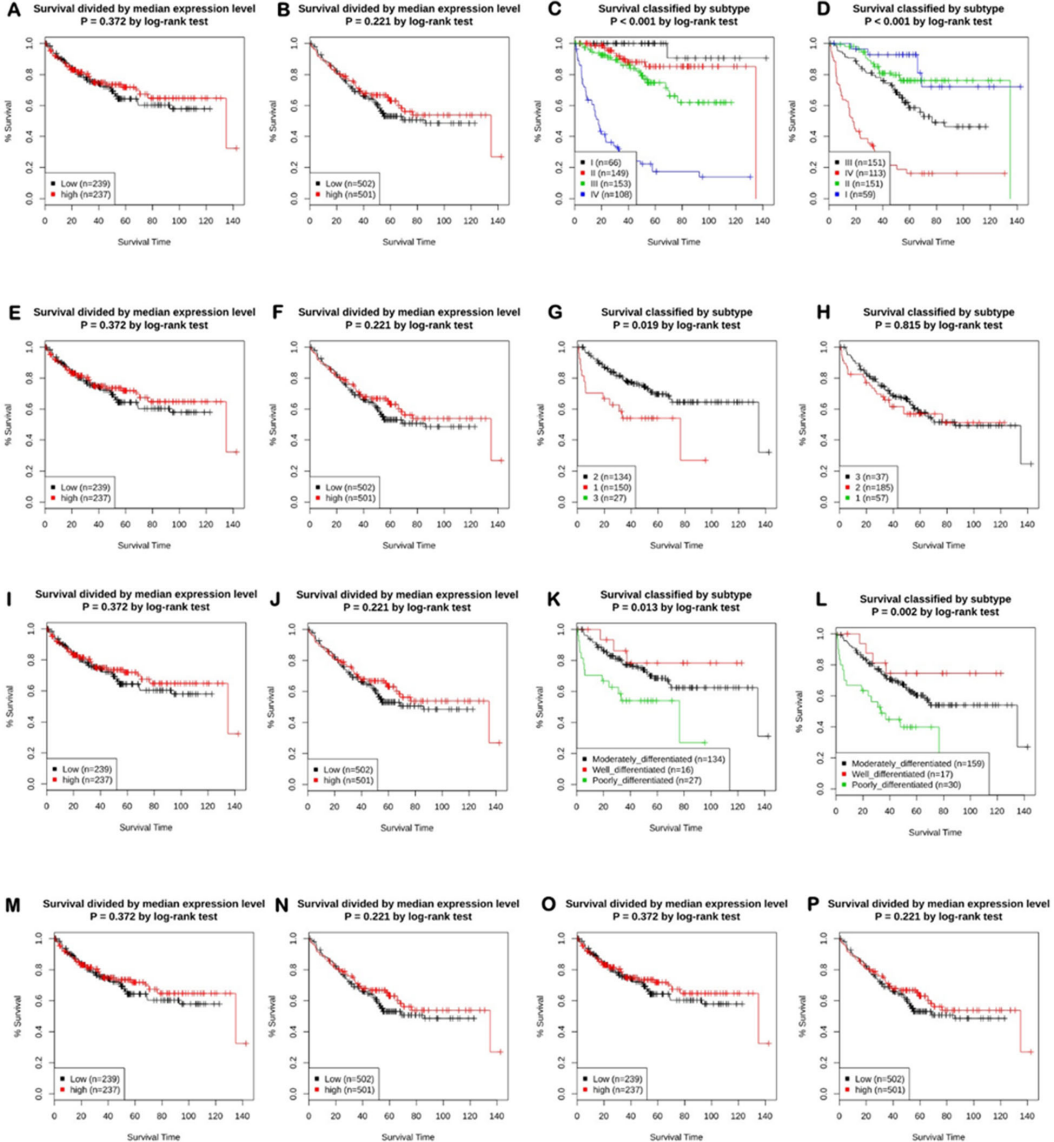
The prognostic outcome of PDE9A in Colon Adenocarcinoma (COAD) patients using GENT2 database. Relation of PDE9A expression level and survival rate based on different variables were analyzed for (**a**) AJCC stage plot divided by median cut off Disease-specific survival; (**b**) AJCC stage plot divided by median cutt off Overall survival; (**c**) AJCC stage plot divided by subtype Disease-specific survival; (**d**) AJCC stage plot divided by subtypes Overall Survival; (**e**) Grade plot divided by median cut off Disease-specific survival; (**f**) Grade plot divided by median cut off Overall survival; (**g**) Grade plot divided by subtype Disease-specific survival; (**h**) Grade plot divided by subtype Overall survival; (**i**) Histology plot divided by median cut off Disease-specific survival; (**j**) Histology plot divided by median cut off Overall survival; (**k**) Histology plot divided by subtypes Disease-specific survival; (**l**) Histology plot divided by subtypes Overall survival; (**m**) Duke stage plot divided by median cutt off Disease-specific survival; (**n**) Duke stage plot divided by median cutt off Overall survival; (**o**) Subtype plot divided by median cut off Disease-specific survival; (**p**) Subtype plot divided by median cut-off Overall Survival

**Fig. 9:**
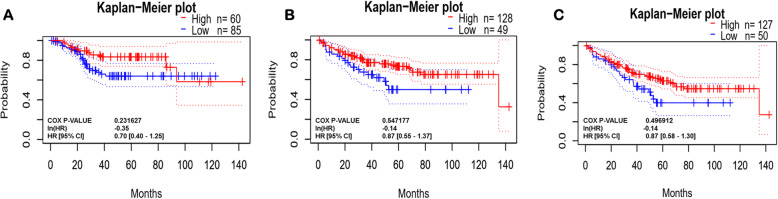
Kaplan-Meier plot of PDE9A from PrognoScan Web server. **a** Disease-free survival; **b** Disease-specific survival; **c** Overall survival

**Fig. 10:**
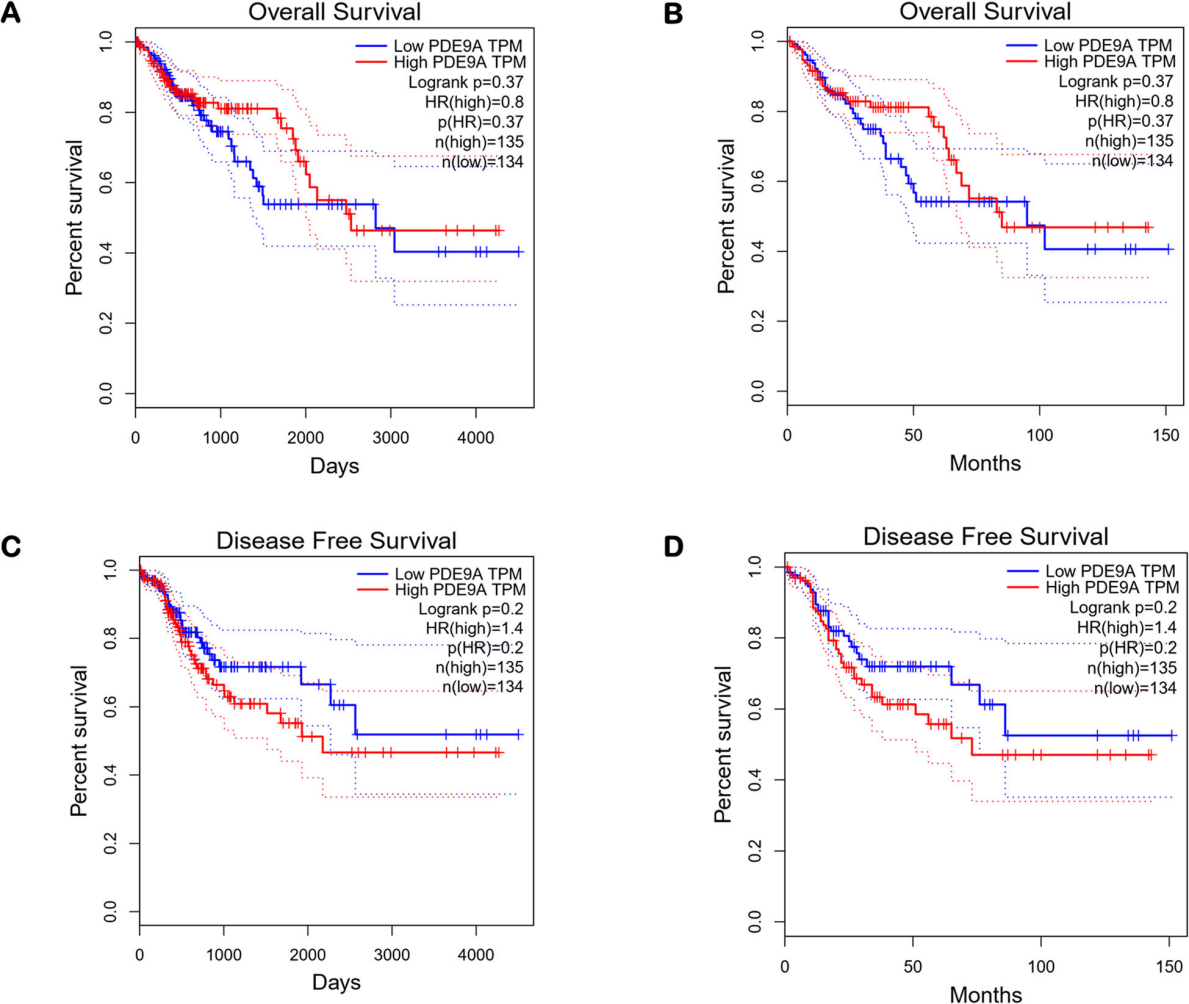
Survival assay of PDE9A in Colon adenocarcinoma (COAD) patient using survival plot from GEPIA database. The survival plots are shown for (**a**) overall survival (Days) and (**b**) overall survival (Months); (**c**) Dease-free survival (Days) and (**d**) Dease-free survival (Months)

The survival plot provides basic statistical concepts to analyze the patient’s survival period on-time event for cancer patients. Survival plot can be Kaplan-Meier plots to visualize survival curves, Log-rank test to compare the survival curves of two or more groups, Cox proportional hazards regression to describe the effect of variables on survival. The most important measures for survival plot in cancer studies include the overall survival (OS) curve, Relapse-free survival (RFS) or Disease-free survival (DFS) curve, and Disease-specific survival (DSS) curve. The relapse-free survival time represents the time between the response of patients to treatment and recurrence of the disease and Disease-free survival is corresponds with the percentage of people in a study who have not died from a specific disease in a defined period. We used different databases namely Gent2 (Fig. 8), PrognoScan (Fig. 9, supplementary Table 3), GEPIA (Fig. 10), UALCAN (Supplementary Fig. 4), OncoLnc (Supplementary Fig. 4), and R2 (Supplementary Fig. 4) to analyze the survival plot or Kaplan-Meier plot for the PDE9A gene against colon cancer. OncoLnc and GENT2 provide the Log-rank test curve and other databases provide Kaplan-Meier plots. The Kaplan–Meier curves disclosed that high expression of the PDE9A gene was related to encouraging conditions in OS (overall survival), RFS (relapse-free survival), and DSS (disease-specific survival) in colon cancer patients. Besides, the Log-rank test curve also depicted that a higher survival rate was detected with high levels of PDE9A expression in colon cancer patients. Conversely, low PDE9A expression is correlated with poor survival, but high PDE9A expression is associated with a higher survival rate. Excluding little exceptions, a low prognostic value was observed by analyzing the survival plot in the case of OS, RFS, and DSS in colon cancer patients. Thus, this study suggests that high expression of the PDE9A gene is positively correlated with a good prognosis in colon cancer patients.

### Interacting network analysis

**Fig. 11:**
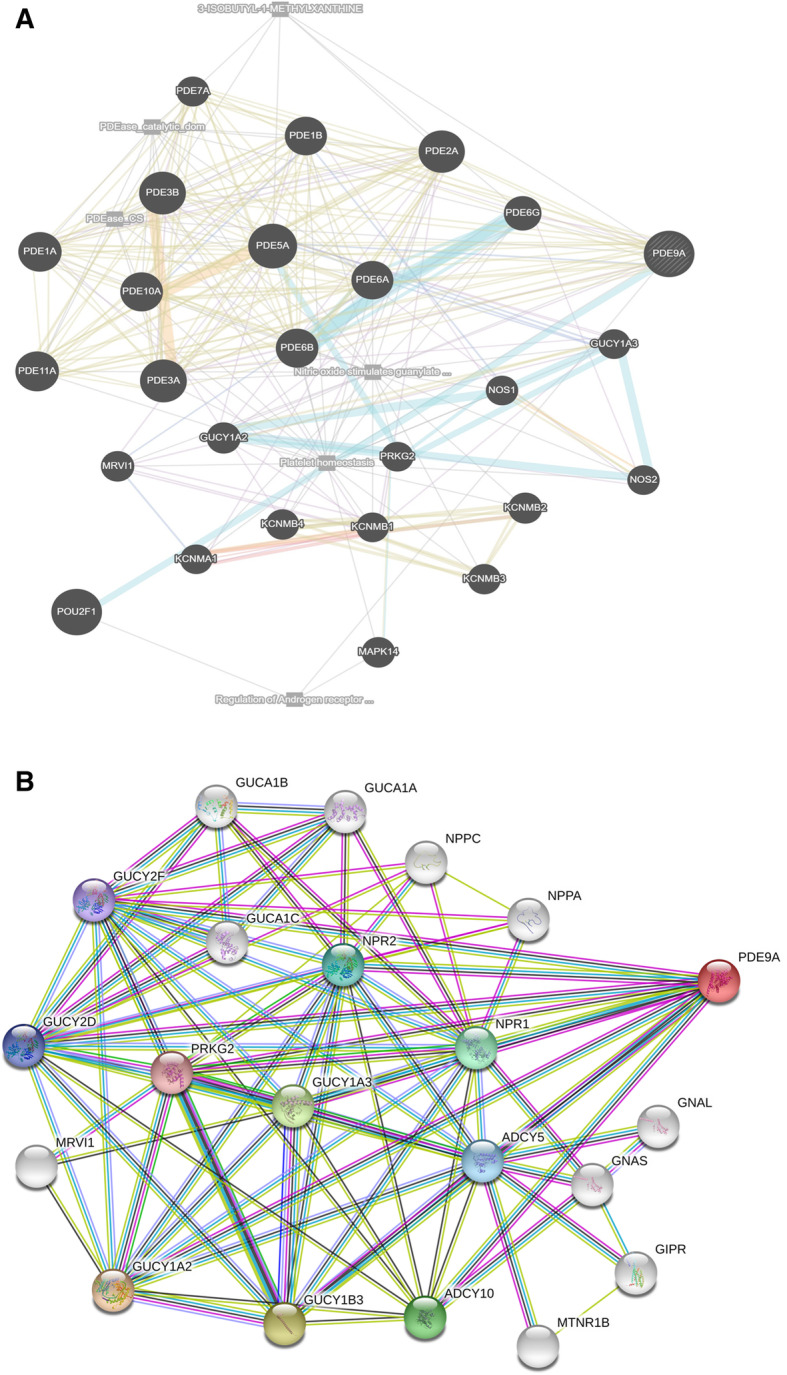
**a** Interaction network of PDE9A gene derived from GeneMANIA. GeneMANIA shows the interactions for genes linked with each other according to physical interaction, genetic interactions, co-expression, pathway, co-localization, and shared protein domain. **b** Protein-protein interaction network for PDE9A gene from STRING database. The interaction network shows that these 20 proteins significantly interact with PDE9A. According to the website, colored nodes represent query proteins and first shell of interactors; white nodes represent second shell of interactors; empty nodes represent proteins of unknown 3D structure; filled nodes represent some 3D structure is known or predicted

To investigate the interaction network, we implemented GeneMANIA and STRING, two different web-based network analysis tools. As we all know that Protein-protein interactions (PPIs) perform a crucial role in cellular functions and biological signaling in all organisms, this may facilitate extensive information regarding interactions and pathways [56]. GeneMANIA provides an interaction network based on the parameters of physical interaction, genetic interactions, co-expression, pathway, co-localization, and shared protein domain. While STRING arranges the physical and functional interactions of genes. To identify the PPIs involving with PDE9A, GeneMANIA provided predicted protein partners are POU2F1, PDE5A, PDE3A, PDE3B, PDE2A, PDE11A, PDE1A, PDE10A, PDE6B, PDE1B, PDE6A, PDE6G, GUCY1A2, KCNMB2, MAPK14, MRVI1, KCNMB4, KCNMB3, PDE7A, KCNMB1, GUCY1A3, PRKG2, NOS1, NOS2, and KCNMA1. KCNMA1, KCNMB1, and KCNMB2 share physical interaction; PDE5A, PDE3A, PDE3B, PDE2A, PDE1A, PDE10A, PDE6B, PDE1B, PDE6A, PDE6G, GUCY1A2, MRVI1, KCNMB4, KCNMB3, PDE7A, GUCY1A3, PRKG2, NOS1, and NOS2 shares co-expression interaction (Fig. 11a). PDE9A contributes consolidated pathways (platelet homeostasis mediated) with MAPK14 which can carry out an important role in colon cancer prognosis. Recent research demonstrates that overexpression of active MAPK14 induces survival autophagy in CRC cells depleted of TP53 and that inhibition of autophagy in such cells enhances the cytotoxic effect [57]. This interaction may lead to PDE9A high expression to a higher survival rate for colon cancer patients. PPI network analysis obtained from the STRING database revealed that PDE9A showed a high confidence interaction (score 0.813) with GUCY1A2 (Guanylate cyclase soluble subunit alpha-2; Has guanylyl cyclase on binding to the beta-1 subunit). It also shares interaction with GUCY1B3, GUCY1A3, ADCY10, NPR1, NPR2, ADCY5, GUCY2D, GUCY2F, PRKG2, GUCA1A, GUCA1B, NPPC, GUCA1C, NPPA, MRVI1, GNAL, GNAS, GIPR, and MTNR1B (Fig. 11b). The network stats are the number of nodes: 21; the number of edges: 89; average node degree:8.48; avg. local clustering coefficient: 0.81 and PPI enrichment *p*-value: < 1.0e-16. These predicted interacting genes of PDE9A may be involved in the regulation of cancer progression and prognosis.

### Co-expression analysis

**Fig. 12:**
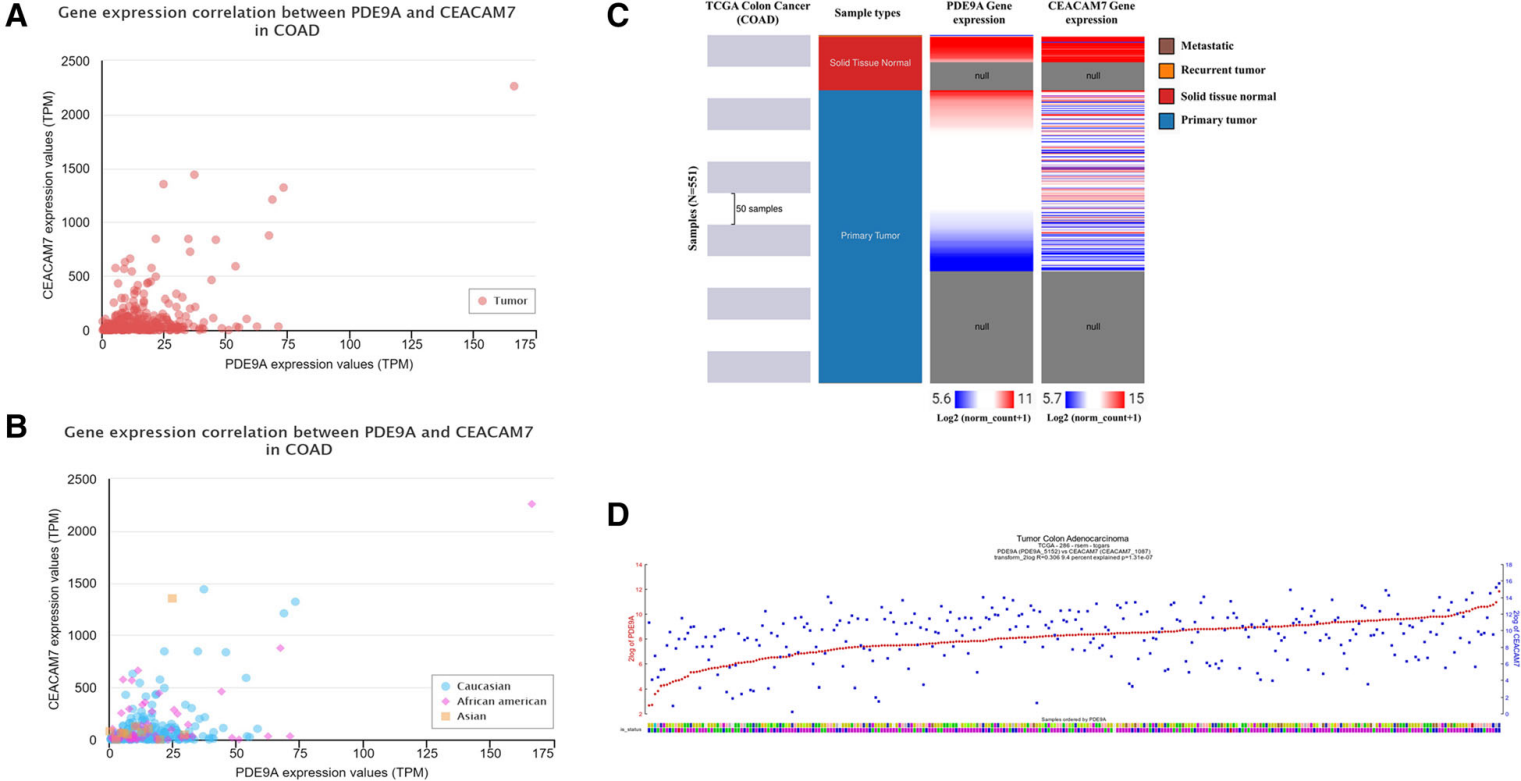
The co-expression profile between the two correlated gene PDE9A and CEACAM7. Gene expression between PDE9A and CEACAM7 using UALCAN database **a** based on tumor; **b** based on patient’s race- Caucasian, African- American, Asian; **c** Correlation heat map of PDE9A and CEACAM7 across sample types in the TCGA colon cancer database using UCSC Xena. **d** The correlated expression of the PDE9A gene with the CEACAM7 gene in Tumor Colon Adenocarcinoma - TCGA - 286 - rsem - tcgars dataset collected from R2 web tool

To identify a co-ordinated expression mediated cancer prognosis of PDE9A, we employed the co-expression profile via UALCAN website. PDE9A shows the highest positive correlation with CEACAM7 in COAD (Pearson CC = 0.54) (Fig. 12a and b). This positive interaction was confirmed by adopting the Oncomine database which ensured that CEACAM7 was also under-expressed in colon cancer tissues. To ensure this positive co-expression pattern between PDE9A and CEACAM7, we also look into TCGA colon cancer patient data via UCSC Xena (Fig. 12c). R2 database was also used to explore the relations of the PDE9A gene with the CEACAM7 gene using correlation statistics. The co-expression between these two genes was identified in Tumor Colon Adenocarcinoma - TCGA - 286 - rsem - tcgars dataset. The analysis reveals r-value = − 0.306; *p*-value = 1.31e-07; *T*-value = 5.415; degrees of freedom = 284 which declares the significance of the correlation in colon cancer sample. Figure 12d shows their positive correlation and the expression of CEACAM7 follows the expression of PDE9A. The figure portrays the expression of both genes in Tumor Colon Adenocarcinoma dataset which also indicates that the increasing PDE9A expression was followed by increasing expression of CEACAM7 in a good way.

## Discussion

Phosphodiesterase 9A (PDE9A) is a protein-coding gene, which catalyzes the hydrolysis of the intracellular second messenger levels cyclic adenosine 3′,5′-monophosphate (cAMP) and cyclic guanosine 3′,5′-monophosphate (cGMP) [18]. cGMP performs crucial roles in cell proliferation, differentiation, and apoptosis via intracellular cGMP signaling [58, 59] and regulates diverse physiological functions covering platelet aggregation [60], neurotransmission [61], and vascular smooth-muscle modulation [62]. Several studies have been reported that dysregulation of PDE activities is associated with diverse tumors and carcinomas like chronic lymphocytic leukemia, glioblastoma, prostate cancer, breast cancer, adrenocortical tumor, etc. [24, 63, 64]. However, the distinct role of PDE9A in CRC is yet to be explored. In the present investigation, the mRNA expression levels, methylation status, prognostic values, PPI interactions, co-expressions in COAD patients were investigated through different databases. Here, we have utilized several bioinformatics databases including Oncomine, UALCAN, GEPIA, GENT2, R2, OncoLnc, PrognoScan, cBioportal, UCSC Xena, GeneCards, GeneMania, and STRING to detect the role of PDE9A in cancer prognosis and progression.

Here, the mRNA expression patterns of the PDE9A in CRC among specific parameters were evaluated adopting Oncomine, UALCAN, GENT2, GEPIA databases. mRNA expression analysis from all these databases demonstrates that PDE9A was conspicuously downregulated in CRC tissues with their corresponding normal tissues. PDE9A expression was downregulated in various colorectal cancer types including Colorectal Adenoma, Colon Mucinous Adenocarcinoma, Colon Adenocarcinoma, Colon Adenoma, Colon Carcinoma, Colorectal Carcinoma, etc. (Fig. 2 and Table 1). Moreover, mRNA expression among various clinicopathological criteria from the UALCAN database also ensured the authenticity that PDE9A was downregulated in all the variables used here (Fig. 5). mRNA expression of cGMP-Specific Phosphodiesterase 9A in various tissues was also reported (Figs. 3 and 7 and Supplementary Fig. 1, 3) [65, 66].

Next, we had scrutinized promoter DNA methylation status through the UALCAN database, where promoter methylation of PDE9A in COAD was attenuated than normal tissues based on sample types, individual cancer stages, patient race, patient gender, patient age, patient weight, tumor histology, and T53 mutation status (Fig. 6). Furthermore, we correlated PDE9A gene expression and DNA methylation heat map in TCGA colon cancer sample types by adopting the UCSC Xena database (Supplementary Fig. 2). Promoter DNA methylation status analysis from both these databases revealed that variations in the DNA methylation status are considered to be associated in the PDE9A gene expression in different stages of CRC. Diversified genetic and epigenetic modifications are responsible to arise CRC among which DNA methylation is thought one of the most influential epigenetic events occurs during the early stages of such oncogenic transformation [67]. Here, we noticed that DNA hypomethylation is correlated with down-regulated gene expression in CRC. Alternatively, in prostate cancer DNA hypermethylation is demonstrated to be associated with upregulated gene expression resembles the diversity of epigenetic regulation [68]. Thus, the analysis of promoter methylation standards and its clinicopathological attributes in CRC may act as a potential biomarker for tracing the tumor [69–71].

Further, we investigated the prognostic relevance of PDE9A in CRC by analyzing the survival plot or Kaplan-Meier plot from different databases like cBioportal, R2, UALCAN, PrognoScan, OncoLnc, GEPIA, and GENT2, etc. (Figs. 8, 9 and 10 and Supplementary Fig. 4). The Kaplan–Meier curves analysis depicted that elevated PDE9A gene expression was associated with favorable conditions in OS, RFS, and DSS. On the contrary, lower PDE9A expression was correlated with a poor survival rate. Several studies have conferred to investigate the prognostic value of various genes and their survival outcomes in CRC to recognize genes that could potentially perform as novel prognostic predictors [72–74]. In Malaysia, survival study and prognostic circumstances for CRC patients were analyzed for effective initial detection and advancements in cancer medication [75]. Therefore, from the survival plot analysis, we can confer that PDE9A expression might be associated with CRC progression and prognosis.

Protein-protein interaction networks were investigated from GeneMANIA and STRING databases, where GeneMANIA facilitates an enormous set of functional association data [76] and STRING arranges the physical and functional interactions of genes (Fig. 11) [55]. GeneMANIA contributes to predicting the PDE9A interaction patterns with other physically interacting proteins. These physical interactions depicted that PDE9A shares consolidated pathways (platelet homeostasis mediated) with MAPK14. MAPK14 had been reported in the progress of irinotecan resistance in HCT116 cells in which TP53 was wiped out that hinders cell proliferation and brings about survival-autophagy in CRC [57]. Such interactions may promote higher PDE9A expression to a higher survival rate in CRC patients. STRING network analysis for PDE9A demonstrated a higher confidence interaction with GUCY1A2. GUCY1A2 displayed a higher level (> = 50%) DNA hypermethylation that resembles a prognostic signature in colon cancer [77]. Besides, GUCY1A2 had been identified as biologically significant to rushing castration-resistant prostate cancer [78]. Thus, PDE9A expression might have significant relevance to compelling CRC that can perform as inhibitors for the identified targets.

For understanding, the molecular mechanism of CRC prognosis and progression with PDE9A expression must be scrutinized. We explored the co-expression analysis of the PDE9A gene adopting UALCAN, R2, and UCSC Xena databases (Fig. 12). The co-expression profile analysis adopting TCGA data through the UALCAN database demonstrated the highest positive correlation with CEACAM7 in COAD. R2 database and TCGA colon cancer patients data from UCSC Xena also ensured the positive correlation between PDE9A and CEACAM7. TCGA data analysis and Oncomine database analysis exhibited that it is under-expressed in CRC. CEACAM7 is a member of the carcinoembryonic antigen (CEA) protein family and encodes a cell surface glycoprotein. Down-regulated gene expression of this gene may be inspected in the colon and rectal cancer [79, 80]. CEACAM7 plays important role in cancer pathology and may provide valuable information to predict promising prognostic markers of CRC and to unravel the molecular mechanism of CRC [73, 81, 82].

In summary, through a standardized data mining process, PDE9A gene expression patterns and methylation status were examined handling openly accessible expression and clinicopathological data. These analyses suggest that PDE9A expression was downregulated in various CRC tissues with their corresponding normal tissues and promoter DNA methylation might have significant relevance with PDE9A expression. Besides, KM plotter analysis revealed prognostic significance in which elevated PDE9A gene expression was combined with positive conditions in OS, RFS, and DSS, alternatively lower PDE9A expression corresponded with a poor survival rate. In addition, PPIs and co-expression analysis demonstrated that PDE9A shares interaction networks with other genes that had a promising role in cancer pathology and may act as a potential biomarker or prognostic marker. Some functional proteins were co-expressed with PDE9A like CEACAM7, a prognostication marker for rectal cancer reappearance. Consequently, it is speculated that PDE9A is a promising prognosis predictor and curative target of CRC.

As the current research centered solely on in silico analysis, a large scale clinical experiment is needed to scrutinize the molecular mechanism of PDE9A in CRC both in vitro and in vivo. Therefore the present study could be validated through multiple wet lab experiment including Gene expression analysis [83], Human Cell Line/Animal Model experiment [84], Gene knockout/knockdown and gene knock-in experiment [85].

## Conclusion

The present research was conducted to explore the role of the PDE9A gene in CRC using bioinformatics analysis methods. We executed a significant onco-informatics analysis to explore the expression profile and various clinicopathological parameters of the PDE9A gene in CRC. Extensive data mining from various publicly available databases revealed that PDE9A was downregulated in various CRC corresponding with their normal tissues. Besides, promoter DNA methylation status, survival plot analysis, PPIs, and co-expression analysis demonstrated that PDE9A has significant prognostic and clinicopathological value in CRC. Thus, PDE9A might have a potential function as a prognostic biomarker or tumor marker.

## Supplementary Information

**Additional file 1: Supplementary Table 01**: Differential expression analysis of PDE9A in COAD based on different variables using UALCAN database. **Supplementary Table 02**: PDE9A promoter methylation level based on different variables in COAD from UALCAN database. **Supplementary Table 03**: The biological relationship between gene expression and survival from PrognoScan database. **Supplementary Fig. 01**: (A + B) Expression of PDE9A across TCGA cancer with tumor and normal samples from UALCAN database, (C) PDE9A expression profile across all tumor samples and paired normal tissues from GEPIA database (Dot plot, each dots represent expression of samples), (D) PDE9A mRNA expression in normal human tissues based on RNAseq, Microarray, and SAGE using GeneCards database. **Supplementary Fig. 02**: (A) Heat map of PDE9A expression and DNA methylation status across TCGA Colon cancer sample types from UCSC Xena genome browser, (B) PDE9A expression in different colon cancer DNA methylation clusters from UCSC Xena. **Supplementary Fig. 03**: Colon cancer tissue subtype profiling analysis for PDE9A gene based on molecular subtype, AJCC stage, Duke stage, Grade, and Histology from Gent2 database. **Supplementary Fig. 04**: The prognostic value of PDE9A expression on colon cancer. Survival plot from UALCAN database (A) Effect of PDE9A expression level on COAD patient survival; (B) Effect of PDE9A expression level and body weight on COAD patient survival; (C) Effect of PDE9A expression level & Race on COAD patient survival; (D) Effect of PDE9A expression level & gender on COAD patient survival; (E) Survival curve from OncoLnc database, and (F) Kaplan–Meier plot from R2 database in Tumor Colon Adenocarcinoma - TCGA - 286 - rsem - tcgars dataset.

## Data Availability

All data generated and analyzed during this study are included in this article including: - Ensembl database https://asia.ensembl.org/; STRING database (https://string-db.org/); geneMANIA https://genemania.org/; R2 platform (http://r2platform.com);OncoLnc (http://www.oncolnc.org/); Gene Expression Profiling Interactive Analysis (GEPIA) (http://gepia.cancer-pku.cn/index.html); Gene Expression database of Normal and Tumor tissues 2 (GENT2) (http://gent2.appex.kr/gent2/); Oncomine database (http://www.oncomine.org/). UALCAN database (http://ualcan.path.uab.edu/index.html; cBioPortal for Cancer Genomics (https://www.cbioportal.org/);

## Abbreviations

AJCC: American Joint Committee on Cancer
cAMP: cyclic adenosine 3′,5′-monophosphate and
cGMP: cyclic guanosine 3′,5′-monophosphate
CEA: carcinoembryonic antigen
COA: Colorectal Adenoma
COA: Colon Adenoma
COAD: colorectal adenocarcinoma
COCA: Colon Carcinoma
COMAD: Colon Mucinous Adenocarcinoma
CAN: copy number alterations
CEAD: Cecum Adenocarcinoma
CRC: Colorectal cancer
CRC: Colorectal Carcinoma
DFS: Disease-free survival
DSS: Disease-specific survival
KM: Kaplan-Meier
lncRNAs: Long non-coding
OS: overall survival
PDE: Cyclic nucleotide phosphodiesterase
PDE9A: phosphodiesterase 9A
READ: Rectal Adenocarcinoma
REMAD: Rectal Mucinous Adenocarcinoma
RFS: Relapse-free survival
SAGE: Serial Analysis of Gene Expression
sDNA: Stool DNA
TCGA: The Cancer Genome Atlas Program
