# Supplementary material for: Prognostic and clinicopathological insights of phosphodiesterase 9A gene as novel biomarker in human colorectal cancer
Source: BMC Cancer. 2021 May 20;21:577. doi: 10.1186/s12885-021-08332-3 (PMC8136133; doi:10.1186/s12885-021-08332-3)
Supplement: Supplementary file 1 — Additional file 1: Supplementary Table 01: Differential expression analysis of PDE9A in COAD based on different variables using UALCAN database. Supplementary Table 02: PDE9A promoter methylation level based on different variables in COAD from UALCAN database. Supplementary Table 03: The biological relationship between gene expression and survival from PrognoScan database. Supplementary Fig. 01: (A + B) Expression of PDE9A across TCGA cancer with tumor and normal samples from UALCAN database, (C) PDE9A expression profile across all tumor samples and paired normal tissues from GEPIA database (Dot plot, each dots represent expression of samples), (D) PDE9A mRNA expression in normal human tissues based on RNAseq, Microarray, and SAGE using GeneCards database. Supplementary Fig. 02: (A) Heat map of PDE9A expression and DNA methylation status across TCGA Colon cancer sample types from UCSC Xena genome browser, (B) PDE9A expression in different colon cancer DNA methylation clusters from UCSC Xena. Supplementary Fig. 03: Colon cancer tissue subtype profiling analysis for PDE9A gene based on molecular subtype, AJCC stage, Duke stage, Grade, and Histology from Gent2 database. Supplementary Fig. 04: The prognostic value of PDE9A expression on colon cancer. Survival plot from UALCAN database (A) Effect of PDE9A expression level on COAD patient survival; (B) Effect of PDE9A expression level and body weight on COAD patient survival; (C) Effect of PDE9A expression level & Race on COAD patient survival; (D) Effect of PDE9A expression level & gender on COAD patient survival; (E) Survival curve from OncoLnc database, and (F) Kaplan–Meier plot from R2 database in Tumor Colon Adenocarcinoma - TCGA - 286 - rsem - tcgars dataset. [file 12885_2021_8332_MOESM1_ESM.docx]

***Original Research Article***

**Prognostic and Clinicopathological Insights of Phosphodiesterase 9A gene as Novel Biomarker in Human Colorectal Cancer**

Tasmina Ferdous Susmi^1¶^, Atikur Rahman^1,2¶^, Md. Moshiur Rahman Khan^1^, Farzana Yasmin^1^, Md. Shariful Islam^3, 4*^, Omaima Nasif^6^, Sulaiman Ali Alharbi^7^, Gaber El-Saber Batiha^8^, Mohammad Uzzal Hossain^5^

^1^Department of Genetic Engineering and Biotechnology, Faculty of Biological Science and Technology, Jashore University of Science and Technology, Jashore-7408, Bangladesh.

^2^Department of Fermentation Technology, School of Biotechnology, Jiangnan University, China.

^3^Department of Reproductive and Developmental Biology, Graduate School of Life Science,

Hokkaido University, Sapporo, 5 Chome Kita 8 Jonishi, Kita Ward, Sapporo, Hokkaido 060-

0808, Japan.

^4^Department of Biology, University of Kentucky, 101 T.H. Morgan Building Lexington KY 40506-022, USA

^5^Bioinformatics Division, National Institute of Biotechnology, Ganakbari, Ashulia, Savar, Dhaka-1349, Bangladesh.

^6^Department of Physiology, College of Medicine, King Saud University [Medical City], King Khalid University Hospital, PO Box 2925, Riyadh, 11461, Saudi Arabia.

^7^Dept. of Botany & Microbiology, College of Science, King Saud University, P.O Box 2455 Riyadh 11451 Saudi Arabia.

^8^Department of Pharmacology and Therapeutics, Faculty of Veterinary Medicine, Damanhour University, Damanhour 22511, AlBeheira, Egypt.

^¶^ These Authors equally contributed to this manuscript.

**Corresponding Author:** Islam Md Shariful, Department of Biology, University of Kentucky, 101 T.H. Morgan Building Lexington KY 40506-022, USA, [sharifbge@uky.edu](mailto:sharifbge@uky.edu)

**Supplementary Table 01:** Differential expression analysis of PDE9A in COAD based on different variables using UALCAN database.

| **Variables** | **Different stages** | **Comparisons** | **N** | **Statistical significance** |
| --- | --- | --- | --- | --- |
| Sample types | Normal  Primary tumor | Normal-Vs-Primary | 41  286 | 3.42E-11 |
| Individual cancer stages | Stage1  Stage2  Stage3  Stage4 | Normal-Vs-Stage1  Normal-Vs-Stage2  Normal-Vs-Stage3  Normal-Vs-Stage4 | 45  110  80  39 | 2.39E-11  1.10E-11  1.43E-10  5.47E-11 |
| Patient’s race | Caucasian  African-American  Asian | Normal-Vs-Caucasian  Normal-Vs-African American  Normal-Vs-Asian | 193  55  11 | 3.73E-11  1.34E-10  5.68E-12 |
| Patient’s gender | Male  Female | Normal-Vs-Male  Normal-Vs-Female | 156  127 | 4.08E-11  2.76E-11 |
| Patient’s weight | Normal weight  Extreme weight  Obese  Extreme obese | Normal-Vs-Normal Weight  Normal-Vs-Extreme Weight  Normal-Vs-Obese  Normal-Vs-Extreme Obese | 70  74  56  10 | 3.55E-11  3.39E-11  6.46E-11  2.30E-09 |
| Patient’s age | 21-40 Yrs.  41-60 Yrs.  61-80 Yrs.  81-100 Yrs. | Normal-Vs-Age(21-40Yrs)  Normal-Vs-Age(41-60Yrs)  Normal-Vs-Age(61-80Yrs)  Normal-Vs-Age(81-100Yrs) | 12  90  149  32 | 3.17E-04  7.84E-11  2.22E-11  1.33E-11 |
| Histological subtype | Adenocarcinoma  Mucinous adenocarcinoma | Normal-Vs-Adenocarcinoma  Normal-Vs-Mucinous-adenocarcinoma | 243  37 | 1.74E-11  1.25E-09 |
| Nodal metastasis status | NO  N1  N2 | Normal-Vs-N0  Normal-Vs-N1  Normal-Vs-N2 | 166  70  47 | 1.25E-11  4.62E-11  1.32E-10 |
| TP53 mutation status | TP53-Mutant  TP53-NonMutant | Normal-Vs-TP53-Mutant  Normal-Vs-TP53-NonMutant | 160  122 | 1.90E-11  8.12E-11 |

**Supplementary Table 02:** PDE9A promoter methylation level based on different variables in COAD from UALCAN database.

| **Variables** | **Different stages** | **Comparisons** | **N** | **Statistical significance** |
| --- | --- | --- | --- | --- |
| Sample types | Normal  Primary tumor | Normal-Vs-Primary | 37  313 | 7.16E-03 |
| Individual cancer stages | Stage1  Stage2  Stage3  Stage4 | Normal-Vs-Stage1  Normal-Vs-Stage2  Normal-Vs-Stage3  Normal-Vs-Stage4 | 50  122  88  41 | 1.95E-01  1.35E-01  1.56E-02  1.19E-02 |
| Patient’s race | Caucasian  African-American  Asian | Normal-Vs-Caucasian  Normal-Vs-African American  Normal-Vs-Asian | 220  61  11 | 2.65E-02  1.41E-03  7.74E-01 |
| Patient’s gender | Male  Female | Normal-Vs-Male  Normal-Vs-Female | 167  144 | 4.98E-03  6.87E-02 |
| Patient’s age | 21-40 Yrs  41-60 Yrs  61-80 Yrs  81-100 Yrs | Normal-Vs-Age(21-40Yrs)  Normal-Vs-Age(41-60Yrs)  Normal-Vs-Age(61-80Yrs)  Normal-Vs-Age(81-100Yrs) | 13  96  165  37 | 5.02E-01  2.26E-03  2.29E-02  9.39E-01 |
| Patient’s weight | Normal Weight  Extreme Weight  Obese  Extreme obese | Normal-Vs-Normal Weight  Normal-Vs-Extreme Weight  Normal-Vs-Obese  Normal-Vs-Extreme Obese | 83  80  66  12 | 9.73E-01  3.88E-03  1.09E-02  1.06E-01 |
| Nodal metastasis status | NO  N1  N2 | Normal-Vs-N0  Normal-Vs-N1  Normal-Vs-N2 | 185  74  50 | 8.14E-02  5.77E-03  1.51E-02 |
| Tumor histology | Adenocarcinoma  Mucinous adenocarcinoma | Normal-Vs-Adenocarcinoma  Normal-Vs-Mucinous adenocarcinoma | 264  44 | 9.05E-03  6.98E-02 |
| TP53 Mutation status | TP53 Mutant  TP53 NonMutant | Normal-Vs-TP53 Mutant  Normal-Vs-TP53 NonMutant | 174  136 | 2.38E-03  1.32E-01 |

**Supplementary Table 03:** The biological relationship between gene expression and survival from PrognoScan database.

| **DATASET** | **ENDPOINT** | **PROBE ID** | **N** | ***P*-VALUE** | **COX *P*-VALUE** | **HR [95% CI^low^ - CI^upp^]** | **ln(HR)** |
| --- | --- | --- | --- | --- | --- | --- | --- |
| GSE12945 | Disease Free Survival | 205593_s_at | 51 | 0.388150 | 0.798772 | 1.17 [0.36 - 3.83] | 0.15 |
| GSE12945 | Overall Survival | 205593_s_at | 62 | - | 0.675850 | 0.84 [0.38 - 1.88] | -0.17 |
| GSE17536 | Overall Survival | 205593_s_at | 177 | 0.449868 | 0.496912 | 0.87 [0.58 - 1.30] | -0.14 |
| GSE17536 | Disease Free Survival | 205593_s_at | 145 | - | 0.231627 | 0.70 [0.40 - 1.25] | -0.35 |
| GSE17536 | Disease Specific Survival | 205593_s_at | 177 | 0.282284 | 0.547177 | 0.87 [0.55 - 1.37] | -0.14 |
| GSE14333 | Disease Free Survival | 205593_s_at | 226 | - | 0.489133 | 0.94 [0.80 - 1.11] | -0.06 |
| GSE17537 | Disease Free Survival | 205593_s_at | 55 | - | 0.466644 | 1.22 [0.71 - 2.10] | 0.20 |
| GSE17537 | Disease Specific Survival | 205593_s_at | 49 | 0.164934 | 0.148261 | 1.85 [0.80 - 4.27] | 0.62 |
| GSE17537 | Overall Survival | 205593_s_at | 55 | 0.015608 | 0.079398 | 1.50 [0.95 - 2.35] | 0.40 |


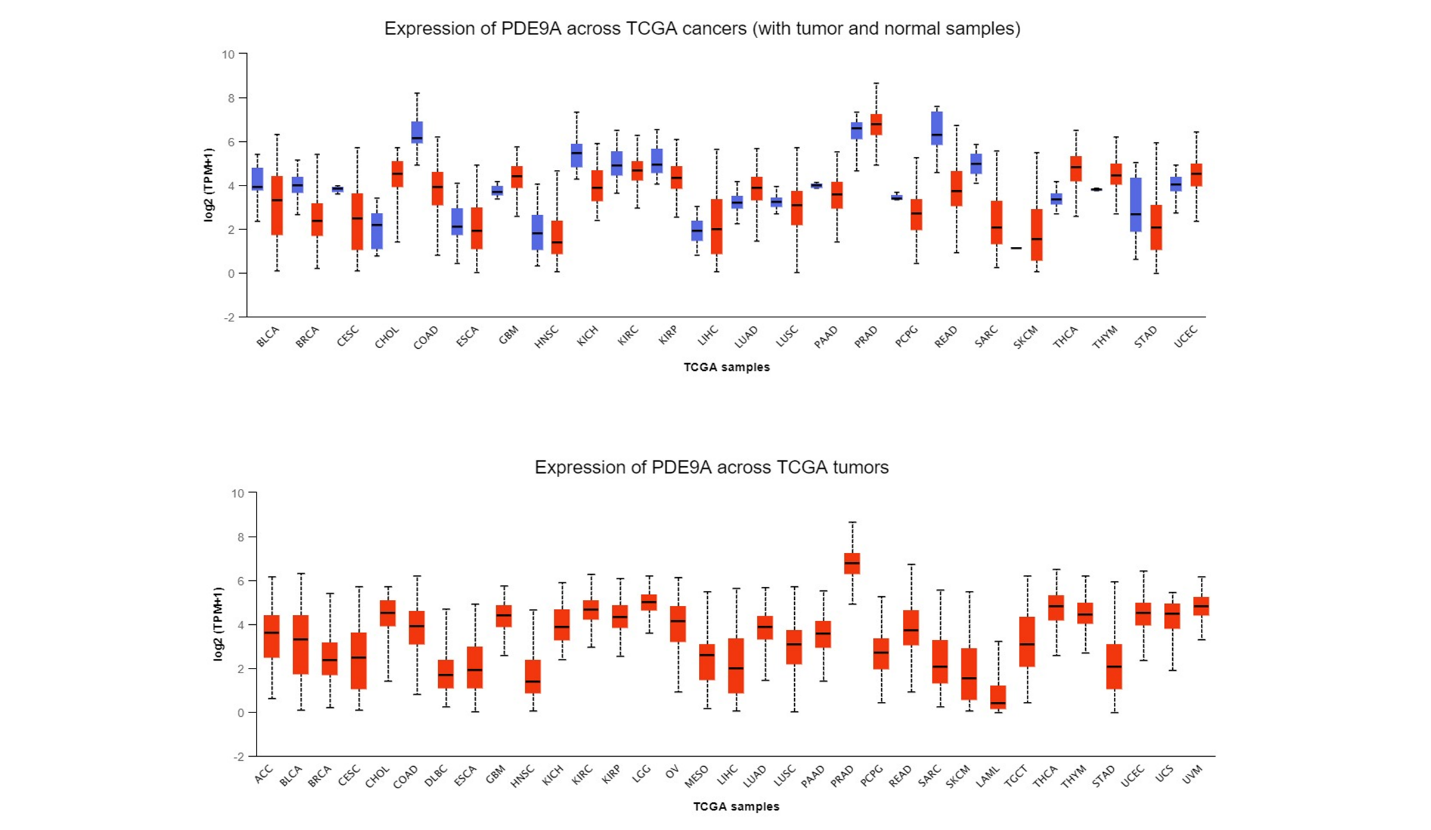


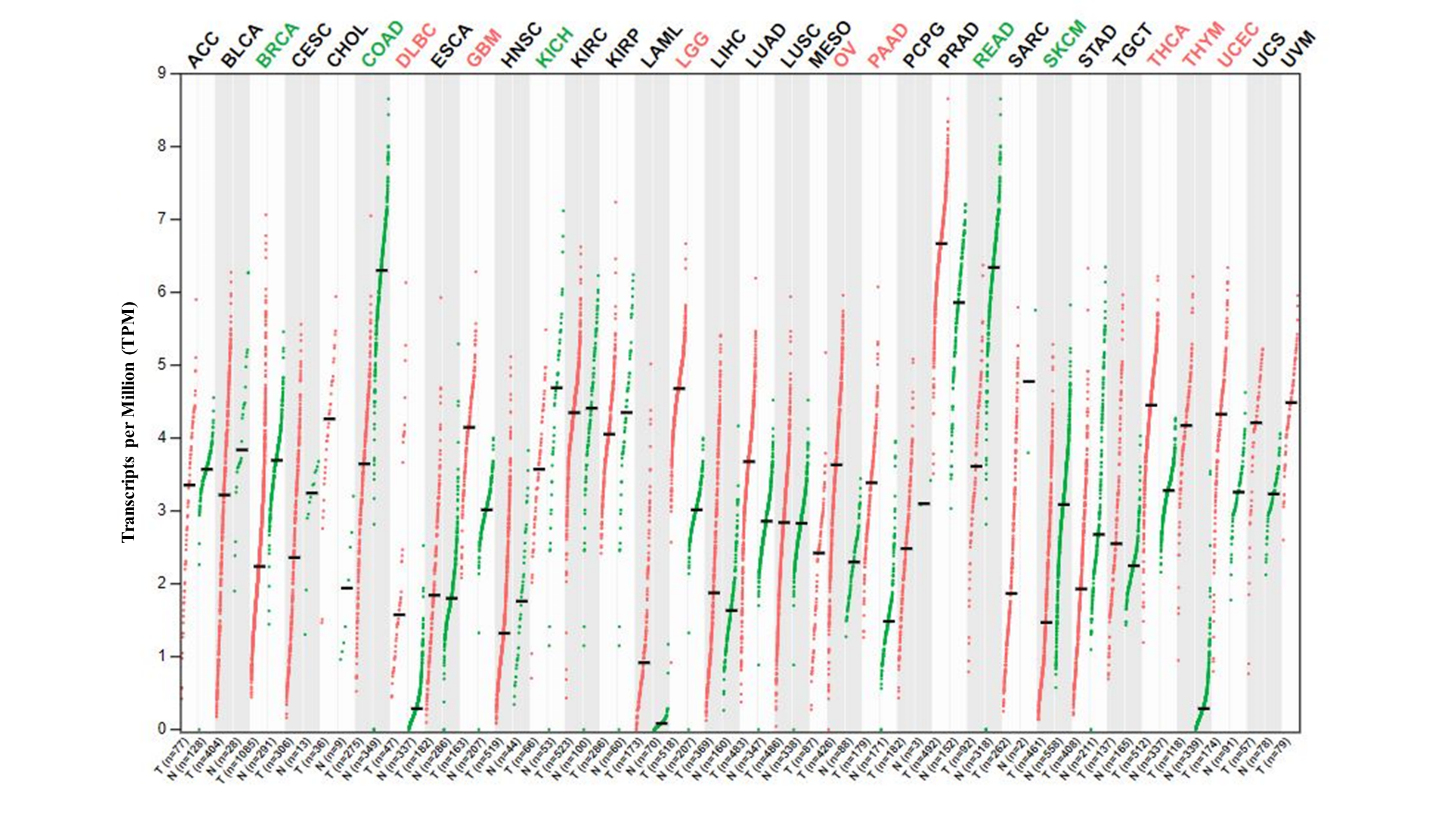




**Supplementary Figure 01: (A+B)** Expression of PDE9A across TCGA cancer with tumor and normal samples from UALCAN database, **(C)** PDE9A expression profile across all tumor samples and paired normal tissues from GEPIA database (Dot plot, each dots represent expression of samples), **(D)** PDE9A mRNA expression in normal human tissues based on RNAseq, Microarray, and SAGE using GeneCards database.
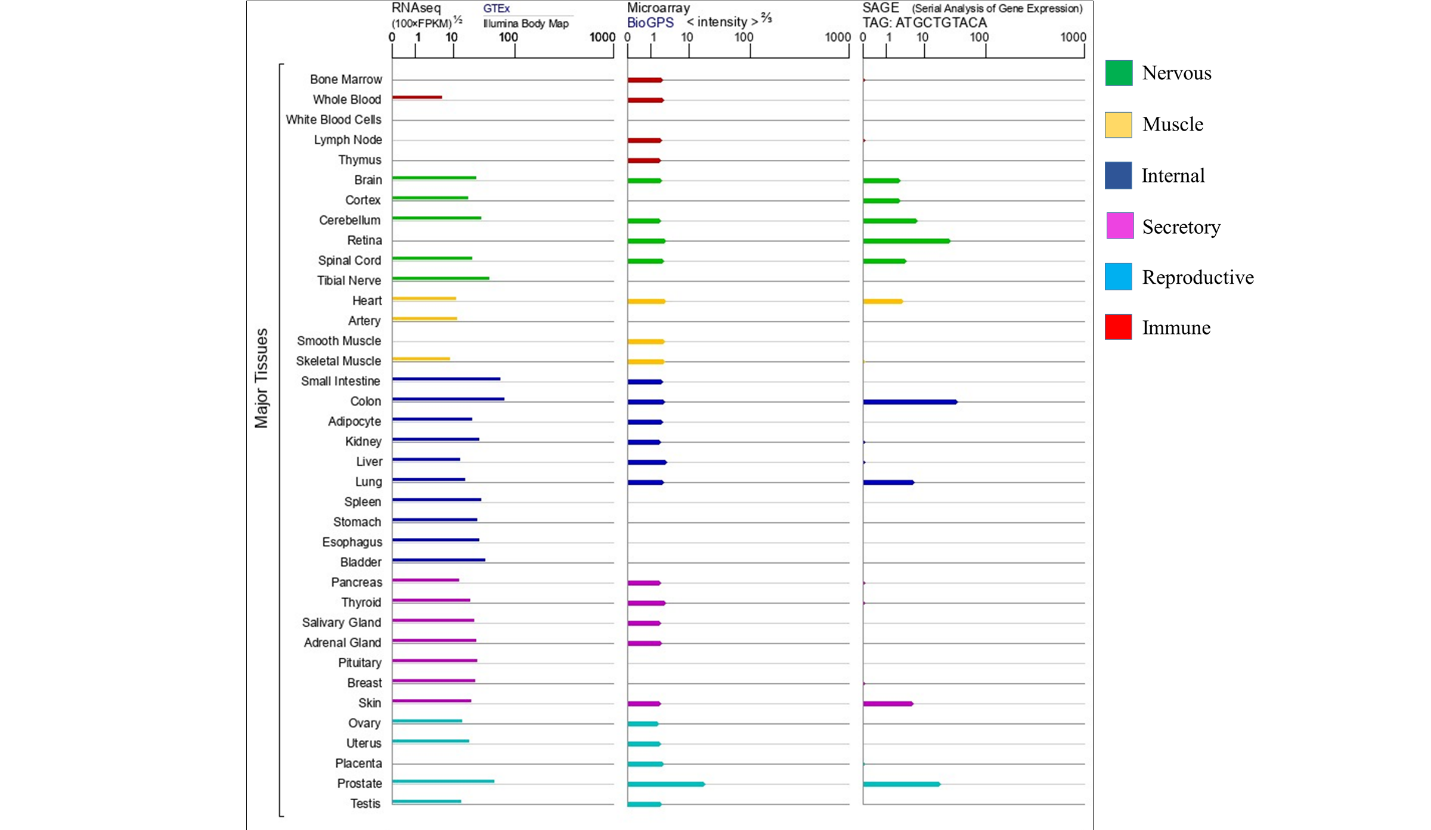


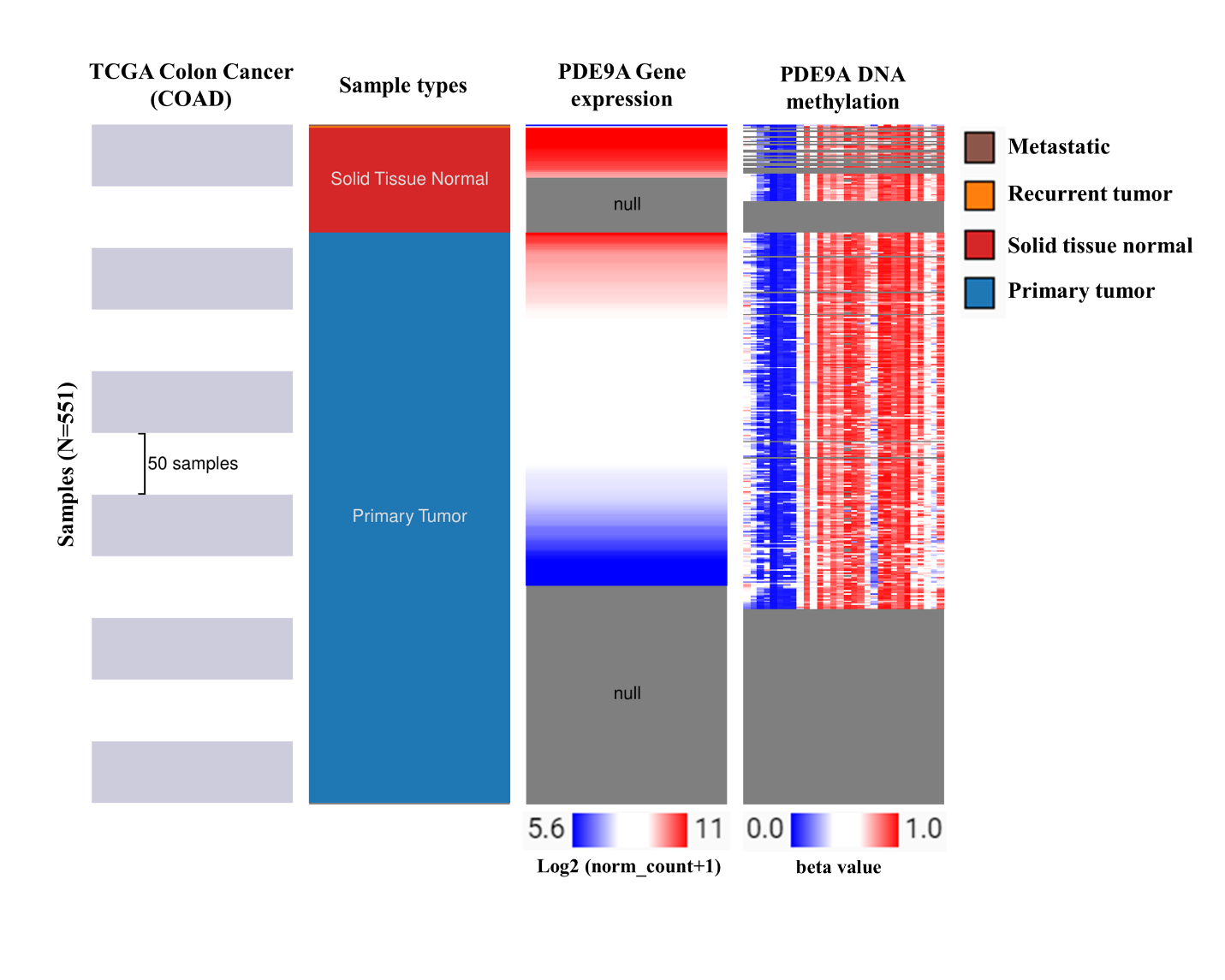


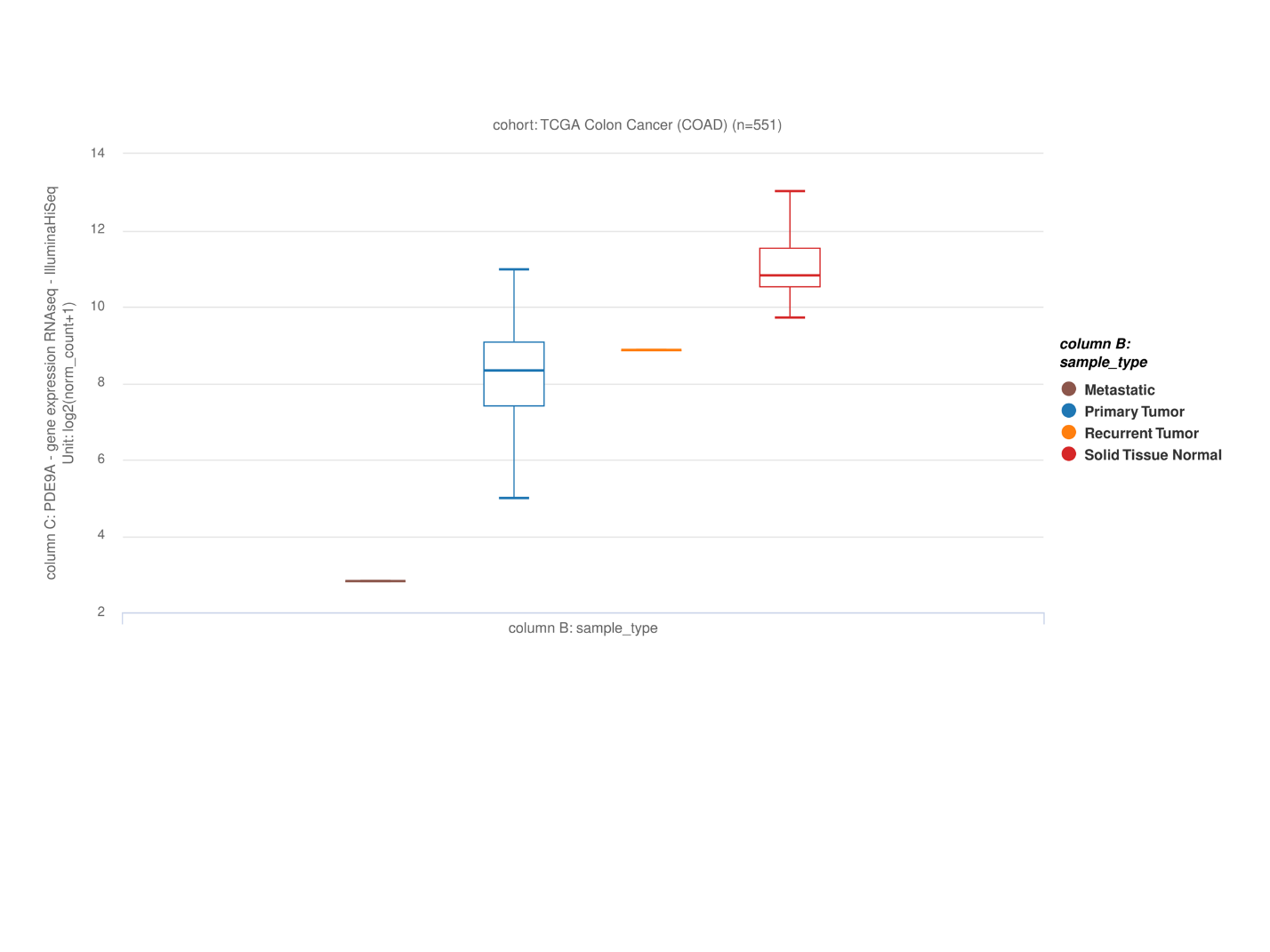


**Supplementary figure 02:** **(A)** Heat map of PDE9A expression and DNA methylation status across TCGA Colon cancer sample types from UCSC Xena genome browser, **(B)** PDE9A expression in different colon cancer DNA methylation clusters from UCSC Xena.

**Supplementary Figure 03:** Colon cancer tissue subtype profiling analysis for PDE9A gene based on molecular subtype, AJCC stage, Duke stage, Grade, and Histology from Gent2 database.
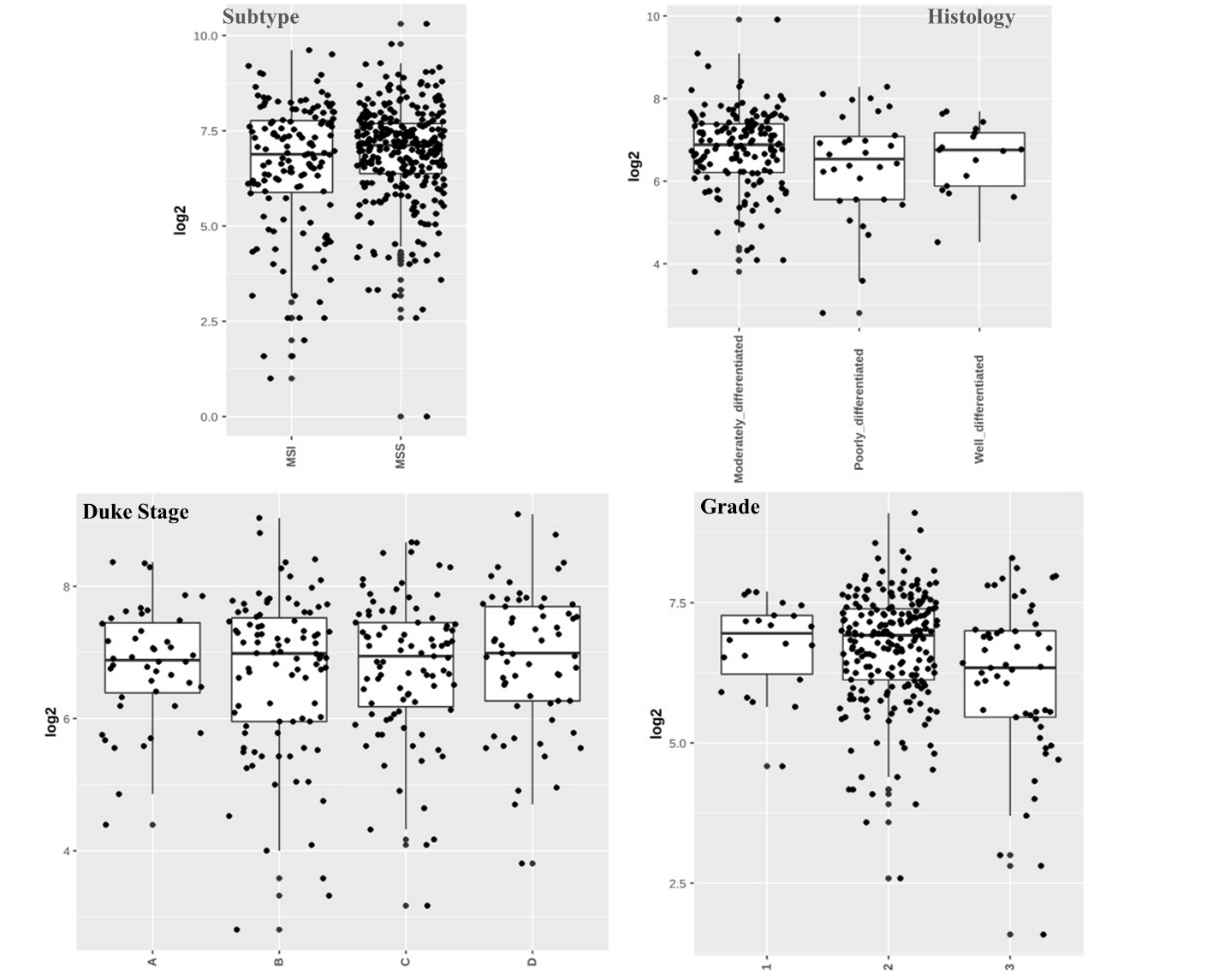

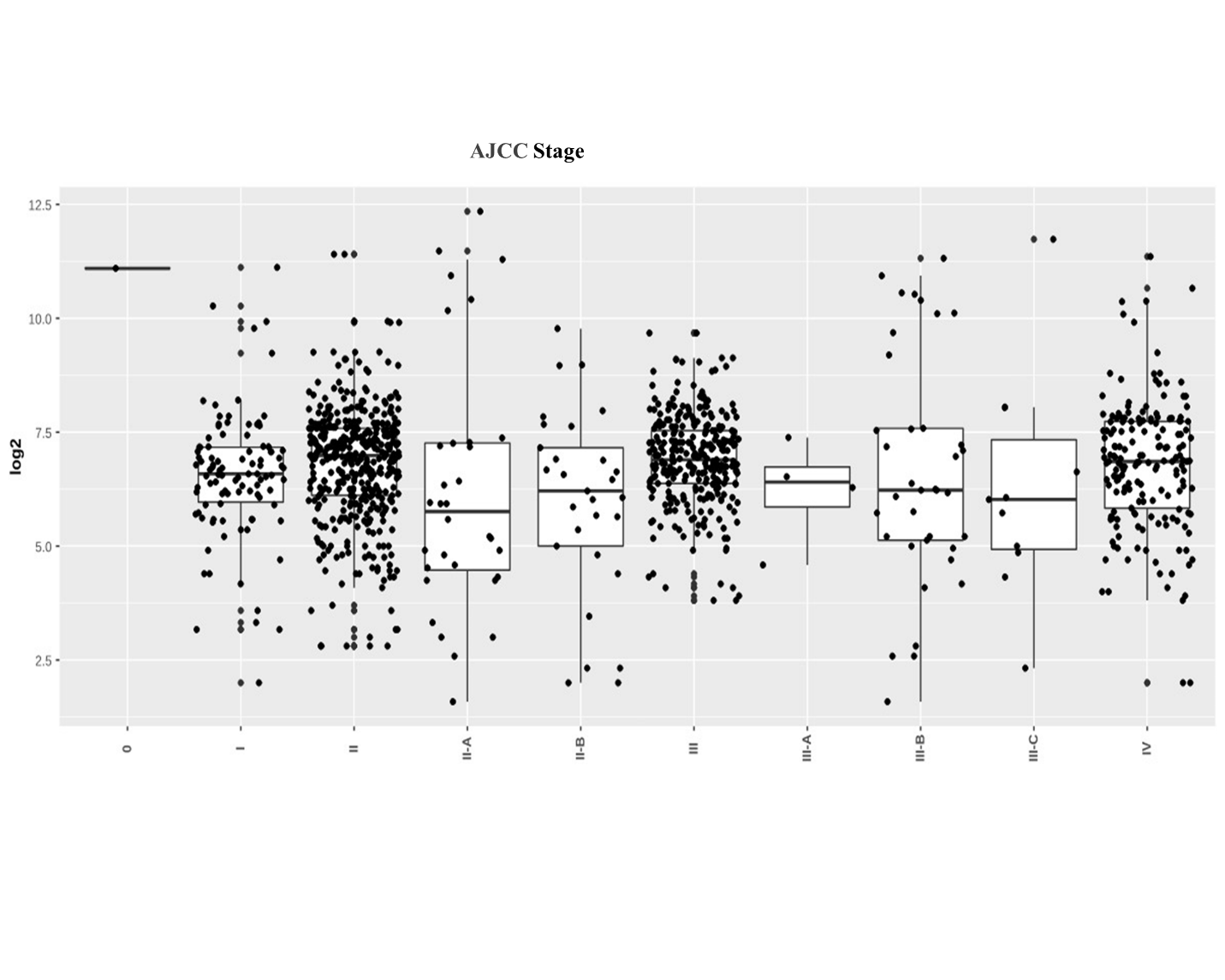


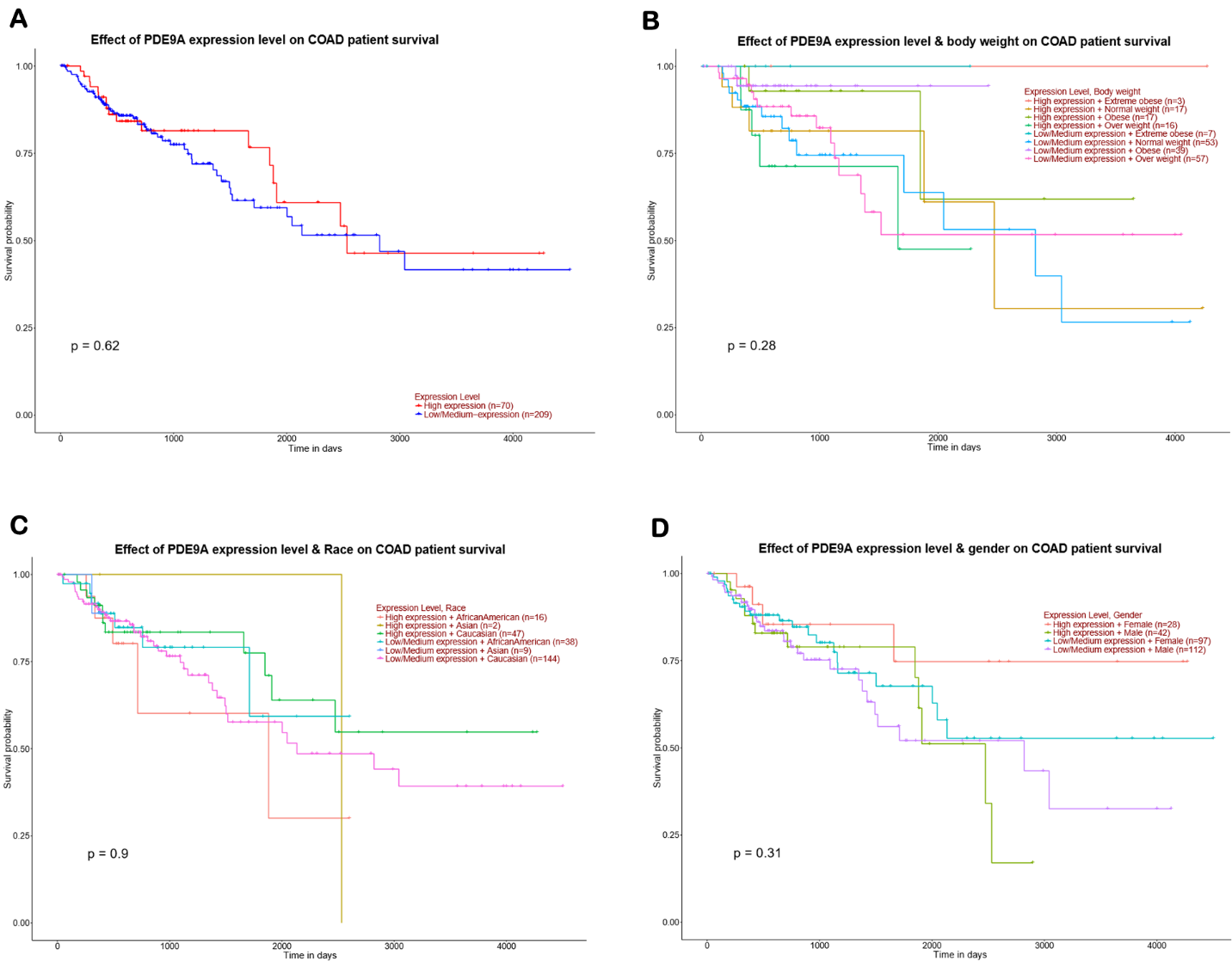

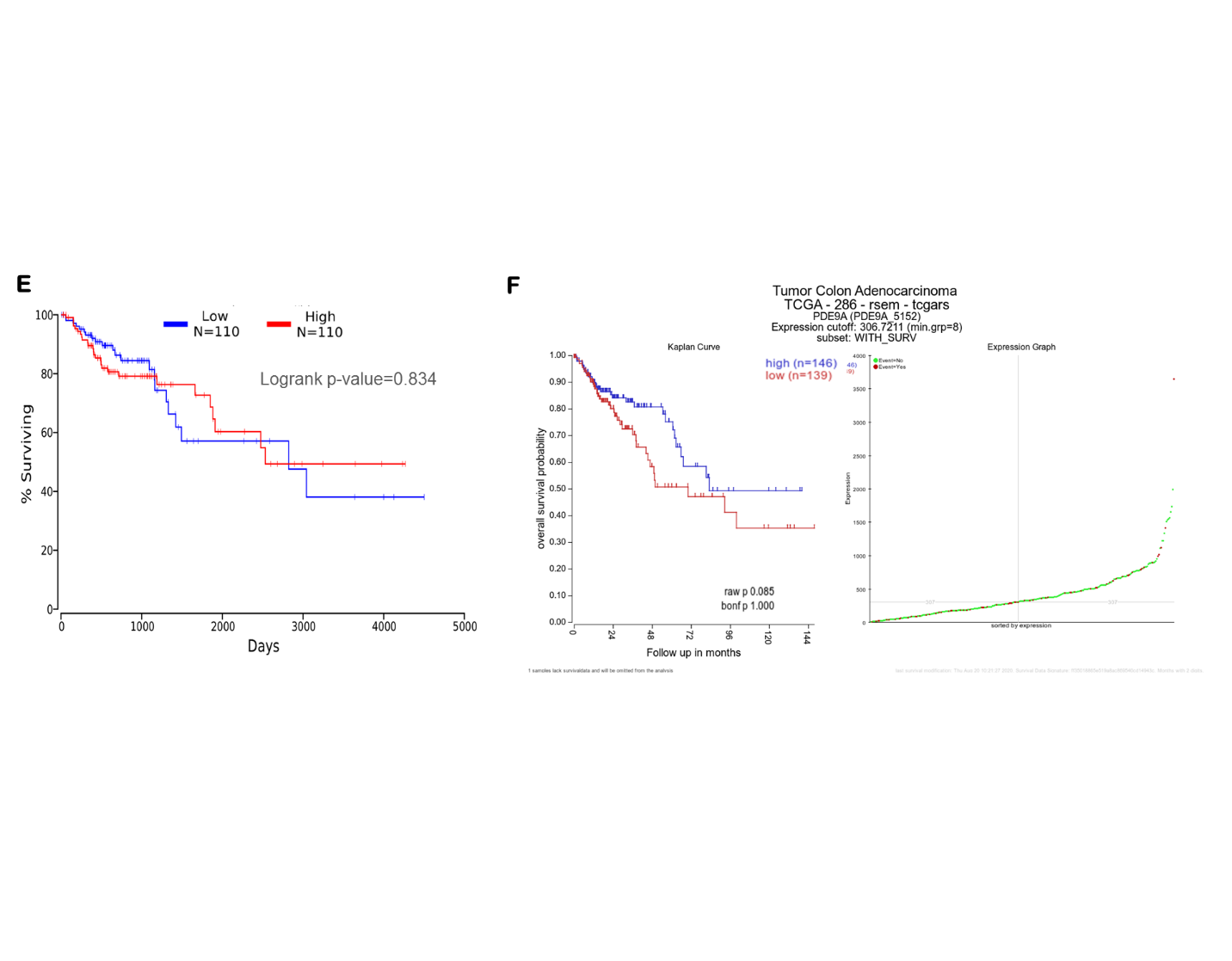


**Supplementary Figure 04:** The prognostic value of PDE9A expression on colon cancer. Survival plot from UALCAN database **(A)** Effect of PDE9A expression level on COAD patient survival; **(B)** Effect of PDE9A expression level and body weight on COAD patient survival; **(C)** Effect of PDE9A expression level & Race on COAD patient survival; **(D)** Effect of PDE9A expression level & gender on COAD patient survival; **(E)** Survival curve from Oncolnc database, and **(F)** Kaplan–Meier plot from R2 database in Tumor Colon Adenocarcinoma - TCGA - 286 - rsem - tcgars dataset.
